# Cooperative stochastic binding and unbinding explain synaptic size dynamics and statistics

**DOI:** 10.1371/journal.pcbi.1005668

**Published:** 2017-07-13

**Authors:** Aseel Shomar, Lukas Geyrhofer, Noam E. Ziv, Naama Brenner

**Affiliations:** 1 Department of Chemical Engineering, Technion, Haifa, Israel; 2 Network Biology Research Laboratories, Lorry Lokey Center for Life Sciences and Engineering, Technion, Haifa, Israel; 3 Russel Berrie Nanotechnology Institute, Technion, Haifa, Israel; 4 Rappaport Faculty of Medicine, Technion, Haifa, Israel; George Mason University, UNITED STATES

## Abstract

Synapses are dynamic molecular assemblies whose sizes fluctuate significantly over time-scales of hours and days. In the current study, we examined the possibility that the spontaneous microscopic dynamics exhibited by synaptic molecules can explain the macroscopic size fluctuations of individual synapses and the statistical properties of synaptic populations. We present a mesoscopic model, which ties the two levels. Its basic premise is that synaptic size fluctuations reflect cooperative assimilation and removal of molecules at a patch of postsynaptic membrane. The introduction of cooperativity to both assimilation and removal in a stochastic biophysical model of these processes, gives rise to features qualitatively similar to those measured experimentally: nanoclusters of synaptic scaffolds, fluctuations in synaptic sizes, skewed, stable size distributions and their scaling in response to perturbations. Our model thus points to the potentially fundamental role of cooperativity in dictating synaptic remodeling dynamics and offers a conceptual understanding of these dynamics in terms of central microscopic features and processes.

## Author summary

Neurons communicate through specialized sites of cell–cell contact known as synapses. This vast set of connections is believed to be crucial for sensory processing, motor function, learning and memory. Experimental data from recent years suggest that synapses are not static structures, but rather dynamic assemblies of molecules that move in, out and between nearby synapses, with these dynamics driving changes in synaptic properties over time. Thus, in addition to changes directed by activity or other physiological signals, synapses also exhibit spontaneous changes that have particular dynamical and statistical signatures. Given the immense complexity of synapses at the molecular scale, how can one hope to understand the principles that govern these spontaneous changes and statistical signatures? Here we offer a mesoscopic modelling approach—situated between detailed microscopic and abstract macroscopic approaches—to advance this understanding. Our model, based on simplified biophysical assumptions, shows that spontaneous cooperative binding and unbinding of proteins at synaptic sites can give rise to dynamic and statistical signatures similar to those measured in experiments. Importantly, we find cooperativity to be indispensable in this regard. Our model thus offers a conceptual understanding of synaptic dynamics and statistical features in terms of a fundamental biological principle, namely cooperativity.

## Introduction

Chemical synapses are sites of cell–cell contact specialized for the rapid transmission of signals between neurons and their targets—muscles, glands or other neurons. The vast majority of synapses in mammals are found in the central nervous system (CNS) where they typically connect the axon of one neuron to the dendrite or soma of another neuron. Structurally, axonal presynaptic compartments are characterized by clusters of synaptic vesicles facing specialized regions of the presynaptic membrane, known as active zones (AZs) [1]; these, in turn, are juxtaposed against electron-dense thickenings of the postsynaptic membrane known as postsynaptic densities (PSD; [2,3]).

The molecular composition of AZs and PSDs is now known in great detail; furthermore, much is now known on the synaptic molecules themselves and on their interactions with other synaptic proteins and membranes. In parallel, recent experiments have provided information on the dynamics of synaptic molecules; these experiments have led to the realization that AZs and PSDs are not static structures but dynamic assemblies of molecules that move in, out and between synapses over time scales of seconds to many hours [4,5]. Such extensive spontaneous dynamics would seem to question the ability of individual AZs, PSDs and synapses in general to maintain their specific sizes (e.g. areas of PSDs and AZs, volumes of spines and presynaptic boutons) and functional properties over behaviorally relevant time scales. Indeed, live imaging studies consistently reveal that instantaneous molecular contents of individual synapses, and by extension, their functional properties, change continuously in manners that are only partially activity dependent (e.g.[4–14,16–20,23–25,27]).

**Fig 1:**
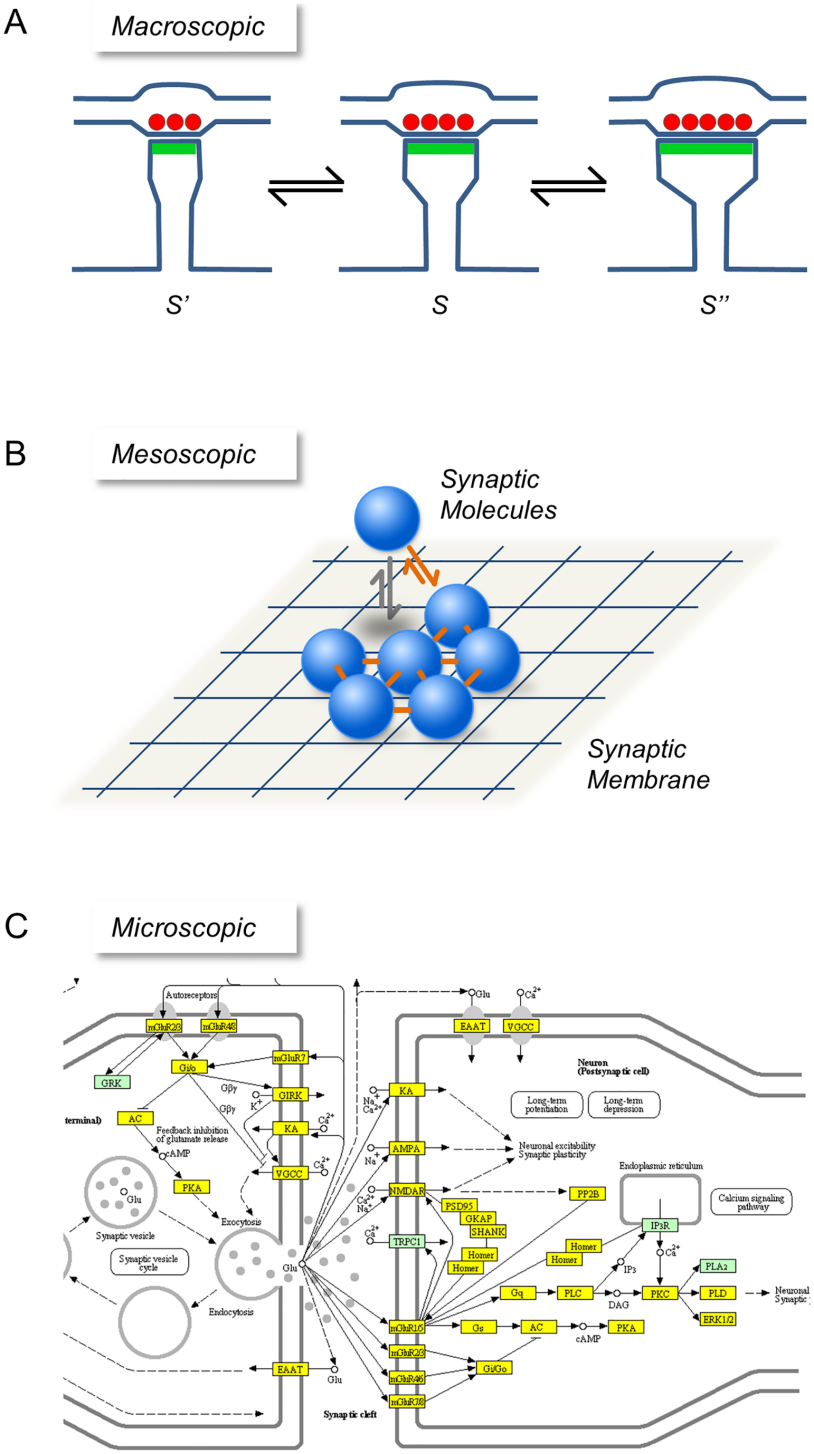
Modeling levels for studying synaptic dynamics. **(A)** Macroscopic level. Here each synapse is characterized by a single variable (S, its momentary size), which changes its value over time according to some statistical process. **(B)**. Mesoscopic level. A small number of central features are considered, in this case the presence of a spatially localized patch of synaptic membrane, synaptic scaffold molecules that continuously bind and unbind to this patch, and the tendency of synaptic molecules to interact among themselves. **(C)** Microscopic level. Here, an attempt is made to model the synapse in great detail, considering the numbers, kinetics and specific interactions of large numbers of synaptic molecules. (Source: http://bioinformatics.charite.de/synsys/; Pathway:Glutamatergic synapse).

The dynamics of synaptic molecules and synaptic properties motivated the formulation of abstract models aiming to describe the properties of individual synapses and synaptic populations [11,14,15,18,19,21–24,26,28,29]. Many of these models were based on low dimensional statistical processes in which each synapse was represented by a single probabilistic variable (e.g. synaptic size; Fig 1A), with causal or deterministic relations emerging at the population level. Somewhat surprisingly, these descriptive models faithfully capture major aspects of synaptic features: the random-like changes in synaptic sizes along time; the stability and skewed shape of synaptic size distributions; the scaling of such distributions in response to changes in network activity as well as other perturbations, and, at the extremes, the dynamics of synapse formation and elimination [14,15,18,19,23,24].

While these descriptive models are quite successful in reproducing many of the aforementioned features, they provide little insight on the principles by which such macroscopic features might emerge from microscopic molecular dynamics within synapses. Conceivably, such insights might be obtained by constructing highly detailed dynamical models, which include all known synaptic molecules, their quantities, their interactions, and the kinetics of all such interactions (Fig 1C). At present, however, in spite of extensive protein-protein interaction databases (e.g. [30]), such models are still not feasible, mainly due to the paucity of data concerning binding affinities and kinetics. More importantly, however, even if such high dimensional, realistic models were feasible, it remains unclear if they are capable of providing intelligible insights on the *principles* by which microscopic molecular dynamics give rise to the macroscopic phenomena mentioned above [31].

In the current study, we describe a *mesoscopic* level exploration that aims to address the following question: Can the spontaneous dynamics exhibited by synaptic molecules give rise to the key features of individual synapses and synaptic populations described above? If so, what are the essential aspects of these dynamics that are necessary for such features to emerge? Mesoscopic level models can be valuable in this respect as they may reveal conceptually tractable principles which would be difficult to glean from highly detailed microscopic models or macroscopic descriptive models [31]. To construct a mesoscopic model, we distilled from the myriad features of synapses and synaptic molecules a small number of key attributes common to practically all synapses, namely a spatially localized patch of membrane, molecules that continuously bind and unbind to this patch, and the strong tendency of such molecules to interact among themselves (Fig 1B). We then use these components to formulate several mesoscopic models of increasing complexity and test their ability to recapitulate major features of synaptic size dynamics, distributions and organization. We show that these macroscopic features emerge naturally from a simple biophysical process based on stochastic binding and unbinding of proteins to spatially confined patches of membrane and to each other, as long as both binding and unbinding have significant cooperative components.

## Results

### Rationale and approach

The emerging view of the synapse as a dynamic molecular assembly implies that at any given moment its size, composition, microscopic organization and ultimately its function, reflect the outcome of myriad processes in which synaptic molecules are assimilated or removed. This applies not only to relatively mobile constituents such as neurotransmitter receptors [5] and synaptic vesicles [7,25, 32,33], but also to synaptic building blocks known as scaffolding molecules. These molecules are generally believed to confer a degree of stability to the sizes and function of synaptic assemblies [4,34]; Moreover, pre- and postsynaptic scaffold molecule contents strongly correlate with functionally important measures of synaptic size, namely AZ and PSD area, respectively (e.g.; [6,16,35–38]). Both PSD molecule content (e.g. [35]) and PSD area (e.g. [36,39]) strongly correlate with dendritic spine volume, which has been repeatedly shown to correlate positively with synaptic strength (e.g. [40–44]); reviewed in [45,46]). Intriguingly, when scaffold molecule contents are followed at individual synapses over time (hours, days) they are found to change considerably (e.g. [6–9,13,16–19,23,25,27]), reflecting in all likelihood, fluctuations in synaptic sizes (e.g. PSD areas, spine volumes) and strengths.

**Fig 2:**
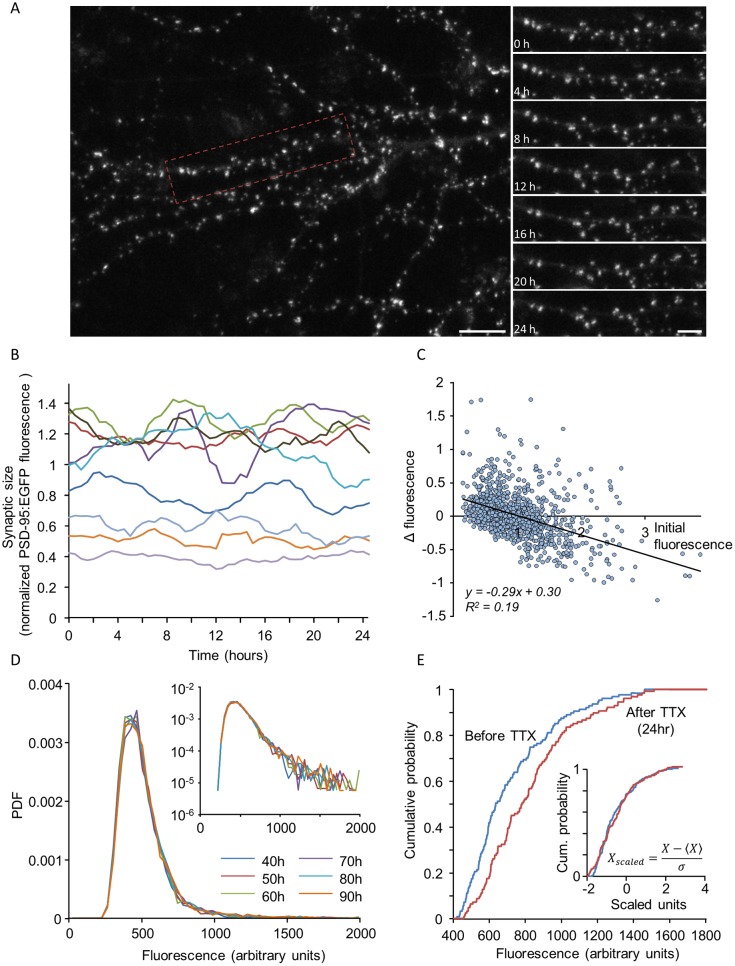
Experimentally measured synaptic size dynamics and statistical properties. **(A)** A portion of the dendritic tree of a rat cortical neuron grown in culture expressing PSD-95 (a major scaffold protein of the postsynaptic densities of glutamatergic synapses) tagged with green fluorescent protein (GFP). The right hand panels show images at 4-hour intervals of a dendritic segment enclosed in the rectangle on the left. The images are maximum intensity projections of 9 sections. Note that in such experiments, measurements were obtained only from dendritic spines that remained well defined and persisted throughout the analysis period. Bars: 10μm (left), 5μm (right). **(B)** 24 hour long traces of fluorescence intensities measured from 10 synapses in rat cortical neurons expressing GFP-tagged PSD-95 (PSD95:EGFP). Data is shown after smoothing with a 5 time point window to decrease the effects of measurement noise and after normalizing to average synapse fluorescence of each cell. **(C)** Changes in the fluorescence of individual synapses as a function of their initial fluorescence. Each dot represents one synapse. ΔF represents the change in fluorescence after 24 hours. Data were normalized as in B. Solid line is a linear regression fit; 1087 synapses from 10 neurons in 5 separate experiments. **(D)** Probability density function (PDF) of PSD-95:EGFP fluorescence values at 10 hour intervals. Inset: same data on semi-logarithmic axes. **(E)** Scaling of synaptic size distributions following the suppression of spontaneous activity. Plots are cumulative distributions of synaptic sizes belonging to a single neuron, before (blue), and 24 hours after (red) pharmacologically suppressing network activity. Inset: Same distributions after scaling. Original images and data plotted in panels A and B were taken from experiments described in [96]; Panels C-E taken from [23].

Experimental studies (e.g. [14,18,19,23]) have given rise to several key observations regarding these fluctuations and their consequences. The first is the observation that whereas sizes of individual synapses fluctuate significantly in time (Fig 2A and 2B), distributions of synaptic sizes in a network are very stable over time (Fig 2D). The second is that these fluctuations are state dependent, namely they depend on the momentary size of the synapse: small synapses tend to grow larger and large synapses to become smaller (Fig 2C), thus supporting the stable population distribution. The third is the observation that these stable size distributions are non-Gaussian and rightward skewed (Fig 2D). Fourth, perturbations of network activity can modify synaptic size distributions while preserving distribution shapes, resulting in the collapse of the different distributions one onto each other when plotted in scaled units (Fig 2E) [18,23,47–50]. Finally, very recent studies based on super-resolution imaging techniques have revealed that PSDs and AZs consist of multiple nanodomains which seem to be dynamic and short lived [51–57].

Can this set of observations be effectively explained by a minimal biophysical model, which views the synapse as the net product of continuous, spontaneous assimilation and removal of their molecular constituents? Below we test this possibility by examining and progressing through a set of biophysical models that differ in the modes of assimilation and removal and, in particular, in the level of cooperativity exhibited by these processes. The feasibility of each model is then assessed by testing the degree to which its outcomes reproduce the experimental observations described above.

Our general framework employs a biophysical model in which synaptic size dynamics stem from binding and unbinding of scaffold proteins to a patch of postsynaptic membrane (Fig 1B). The modeling platform is based on a representation of the postsynaptic membrane as a matrix composed of *M* potential binding sites for synaptic scaffold molecules. Synaptic size at any given time is then estimated as the number of occupied sites, that is, the number of scaffold molecules bound to the matrix. Scaffold molecule binding and unbinding are in principle stochastic events characterized by probabilities per unit time. Consequently, the number of molecules binding to the matrix per unit time depends on the binding probability and on the number of vacant sites. Similarly, the number of molecules dissociating per unit time depends on the unbinding probability per unit time and on the number of bound molecules (= occupied sites). In this stochastic description, the binding and unbinding of proteins result in temporal fluctuations in synaptic size, i.e. in the number of molecules bound to the matrix. An ensemble of synapses is represented by multiple realizations of this stochastic process, and thus a distribution of synaptic sizes emerges across a population of synapses modeled in this fashion.

In the continuum approximation, where fluctuations are neglected, binding and unbinding are described by rate equations. On average, the change in synaptic size is then the outcome of the net effect of these two processes, and average synaptic size *S* follows the continuous equation:

$$\frac{dS}{dt}=k_{on}\left(M-S\right)-k_{off}S$$ (1)

where *k*_*on*_ and *k*_*off*_ are the rate coefficients, the continuous analogs of the binding and unbinding probabilities per unit time, respectively. In general, the binding and unbinding rates are not necessarily constant, and may depend on interactions between the binding molecules; we consider below several cases of such interactions. In what follows, we use the continuous equation to represent the interactions in a compact way and to identify steady states at the level of synaptic populations. However, as we were primarily interested in studying fluctuations and their effects, the results below were mainly derived from discrete numerical simulations based on the stochastic analog of Eq (1). In such simulations, matrices with dimensions of 50x50 were used, with these dimensions loosely derived from measurements of average PSD diameter (360-400nm) and the granularity imposed by widths of canonical PSD molecules and glutamate receptors (5-20nm) [3,58–60]. Monte Carlo simulations were used to determine the occurrence of binding and unbinding events, using a random number generator and the probability per unit time of the relevant event and the current state of binding sites in the matrix (further details are given in the Methods section). Whenever possible, numerical simulation results were compared to continuum approximations, or to the master equation and its solutions (for a review on the relation between continuum and stochastic descriptions, see [61]).

### The Langmuir model: Independent binding and unbinding

**Fig 3:**
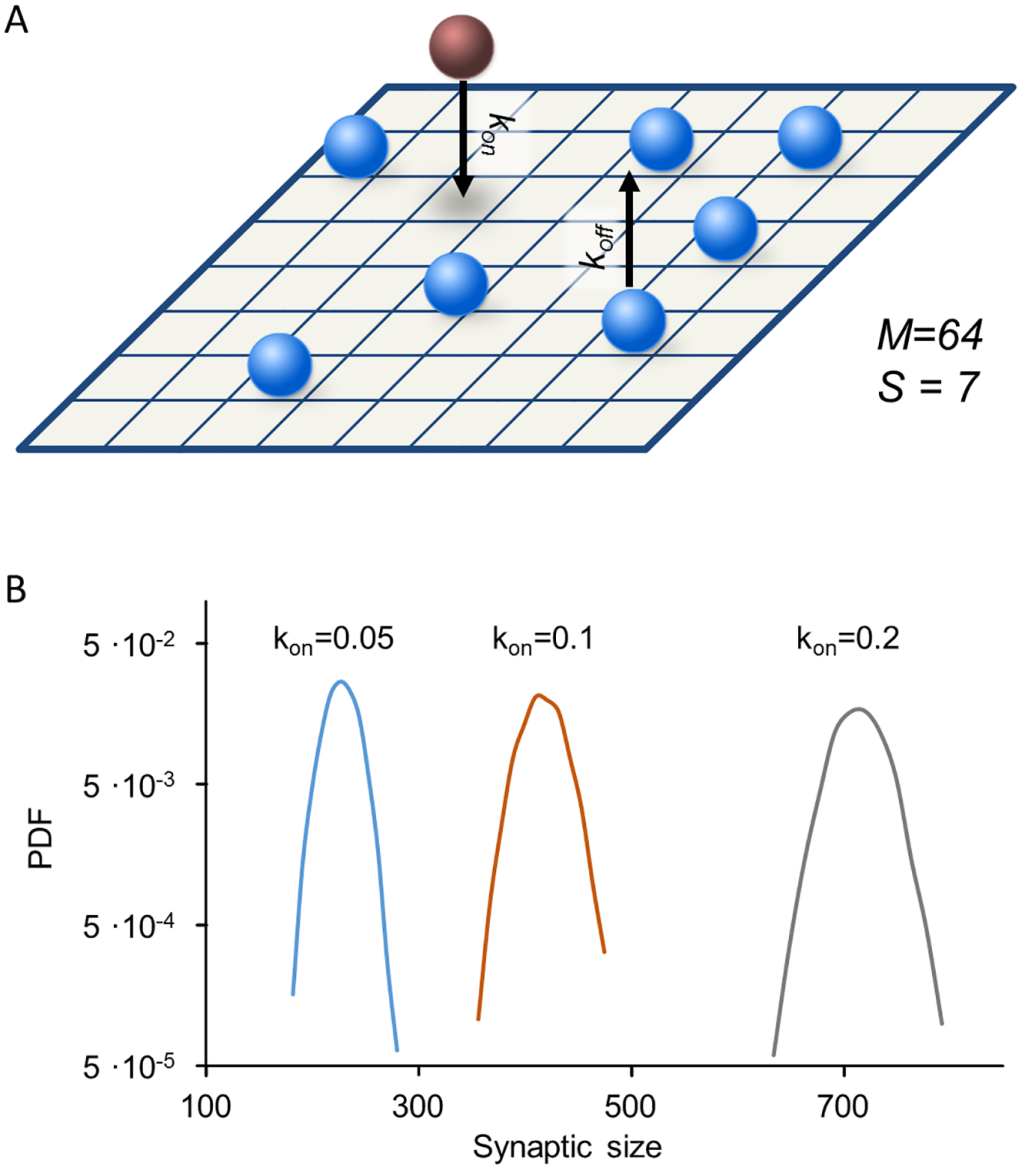
Synaptic size distributions for the Langmuir model. **(A)** Illustration of independent binding and unbinding from the matrix. **(B)** Synaptic size distributions (semi-logarithmic scale) obtained from the Langmuir model for three values of *k*_*on*_, with *k*_*off*_ set to 0.5. Parabolic shape of the curves corresponds to Gaussian-like distributions. Simulated data for 3,500 synapses. See Methods for the rest of the simulation parameters used here.

In the simplest model of binding and unbinding, the rate coefficients *k*_*on*_ and *k*_*off*_ in Eq (1) are constant in space and time: *k*_*on*_ = α, k_off_ = β. In terms of individual molecular events, this implies that scaffold proteins bind to and unbind from the matrix independently from each other and that there are no interactions between molecules (Fig 3A). This model is known in physical chemistry as the Langmuir adsorption model and is used to describe the kinetics of gas adsorption and desorption on a solid surface where interactions between molecules are negligible [62].

Stochastic simulations of the Langmuir model show that the distribution of synaptic sizes in a population is symmetric and well approximated by a normal (Gaussian) distribution (Fig 3B). This is expected in light of the Central Limit Theorem, which states that the sum of a large number of independent, identically distributed random variables is approximately normally distributed, regardless of underlying variables. Since site occupancies are independent random variables, and since synaptic size is a sum of thousands of such variables (2,500 in the simulation), its distribution will be indistinguishable from a normal distribution. These simulation results are corroborated by direct solutions of the master equation in the Fokker-Planck approximation (S1 Appendix, Section 2.2).

As mentioned above, one hallmark of synaptic populations is the broad and highly skewed distribution of their sizes (e.g. Fig 2D). This observation rules out a model of independent binding and unbinding of scaffold molecules at the synaptic site, not a surprising conclusion in light of the multitude of interactions among synaptic proteins. This being so, we next examined whether molecular interdependency might defeat the Central Limit Theorem and give rise to the skewed distributions observed experimentally.

### Cooperative models

Cooperativity is a key organizing principle in biology that represents a fundamental mechanism for accomplishing molecular interdependencies [63–69]. We here define cooperativity as the dependence of the binding affinity of one molecule on the state of the matrix in terms of other molecules already bound to it. In its most simple form, cooperativity could stem from the fact that many (if not all) synaptic molecules have multiple interaction sites through which they are capable of interacting simultaneously with other synaptic molecules; consequently, the presence of other molecules already bound to the matrix may increase the probability of an unbound scaffold molecule to bind as well. Conversely, their presence would be expected to reduce the probability of dissociating from the matrix due to a multiplicity of interactions with neighboring molecules. To examine whether this or other forms of cooperativity can give rise to the experimentally observed synaptic size dynamics and statistical features, we introduce unidirectional and then bidirectional cooperativity into the model as described next.

### The Contact Process: Cooperative binding, independent unbinding

A well-studied model of cooperative binding, which appears to extend the Langmuir model only slightly, is the Contact Process [70,71]. In this model, binding proceeds with a probability that increases with the number of occupied neighboring sites such that

$$k_{on}=\lambda_{on}\chi$$ (2)

**Fig 4:**
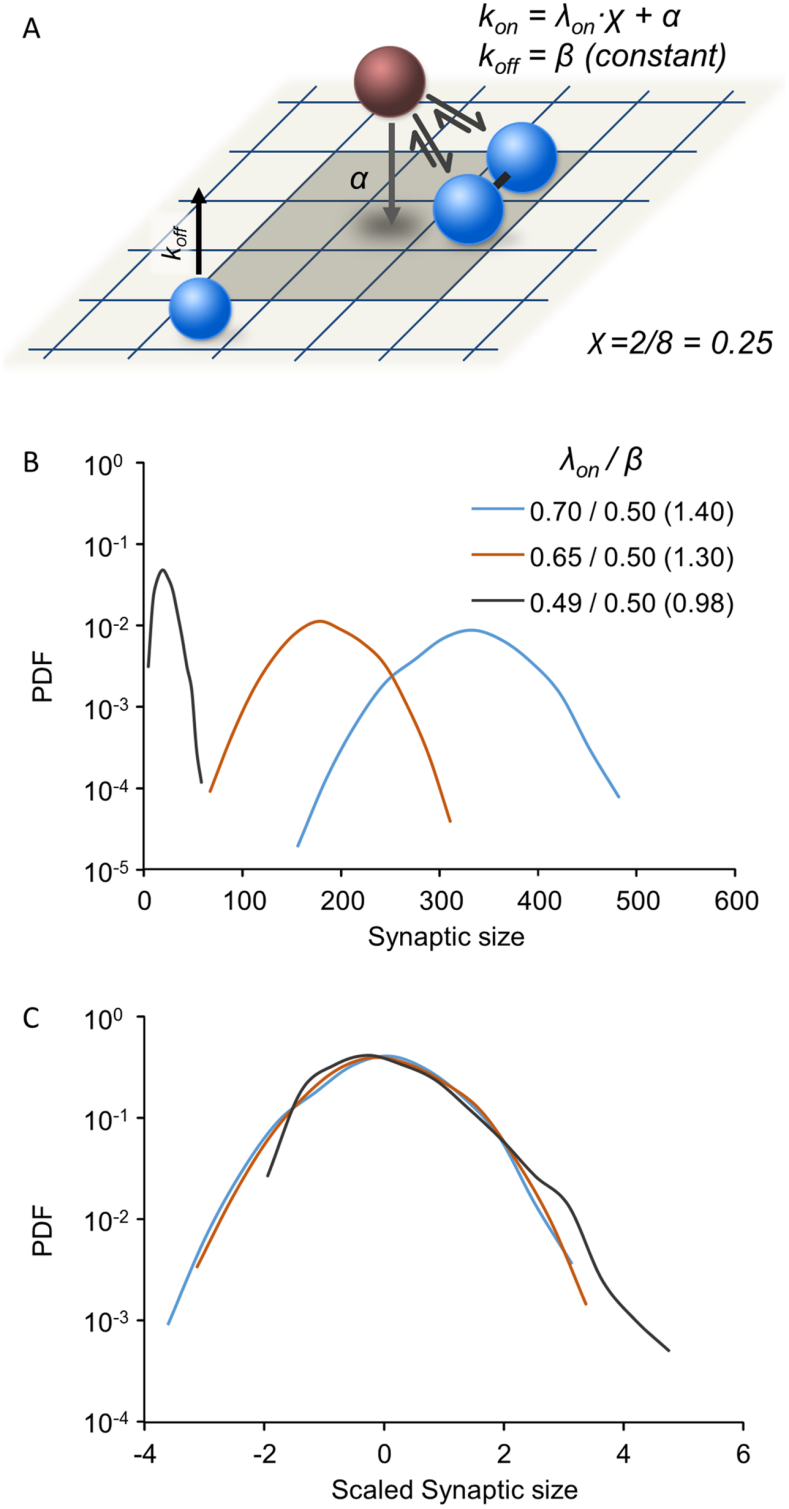
Synaptic size distributions in the Contact Process model. **(A)** Illustration of the Contact Process: Binding is cooperative with a rate that increases with the fraction of occupied neighboring sites (representing interactions with nearby bound molecules) whereas the unbinding rate is a constant, β, insensitive to the numbers of occupied neighboring sites. Binding is also affected by a small, constant and non-cooperative component (*α*) representing weak, non-specific binding to the matrix. **(B)** Synaptic size distributions for different λ_on_/β ratios. All distributions were determined numerically through simulations (3500 synapses, 1500 time steps; see Methods for further details). Note the semi-logarithmic scale. **(C)** Same distributions as in (B) after scaling by subtracting the mean and dividing by the standard deviation. See Methods for the rest of the simulation parameters used here.

Here *λ*_*on*_ is a constant that indicates cooperative binding strength (maximal value of *k*_*on*_) and *0<χ<1* is the fraction of occupied nearest neighbors (with the definition of nearest neighbors depending on matrix geometry as illustrated in Fig 4A). Specifically this implies that the probability of a molecule to bind the matrix at a given site increases if that site has neighboring sites that are already occupied. In contrast to binding, unbinding in the Contact Process is not cooperative and occurs independently from occupancy, consequently, *k*_*off*_ = β. The introduction of cooperative binding changes the behavior of the model dramatically, and results in a transition between two qualitatively different phases. In the supercritical phase, (*λ*_*on*_/β is larger than a threshold critical value), the system approaches a stationary state. The resulting distribution of synaptic sizes in this phase still converges to a normal distribution (See S1 Appendix, S4 Fig). In the subcritical phase (*λ*_*on*_/β is below this threshold), all realizations fall into an absorbing state in which all sites are vacant. A similar model was previously used to address the question of how synapses persist even though their molecular constituents continuously enter and leave the synaptic assembly [22]. It was found that this model extends the expected lifespan of the synaptic assembly relative to the timescales of single molecule binding and unbinding; yet, as expected, assembly sizes ultimately collapse to zero.

To drive the system away from this absorbing state, that is, to obtain steady states at which assemblies have non-zero sizes, an additive constant term *α* can be incorporated into the binding rate, such that

$$k_{on}=\lambda_{on}\chi +\alpha$$ (3)

Biophysically, *α* represents low affinity binding to unoccupied matrix sites (Fig 4A), serving, at the extreme, to seed synapse formation on an empty matrix. The additive component *α* thwarts the collapse into the absorbing empty state, resulting in stable limiting distributions. Unfortunately, as the stochastic simulations show, when *α* is large and dominant, limiting distributions of synaptic sizes become approximately Gaussian; this might be expected, as a major contribution comes from independent binding. Conversely, if *α* is relatively small, slightly skewed distributions can be found, but only for small values of *λ*_*on*_/β (Fig 4B and 4C). However, in this case, the mean synaptic size is very close to zero (Fig 4B).

Although mean synaptic size in our simulations has no particular biological meaning, the very low occupancy fraction found at steady states obtained by adding *α* to the Contact Process, would seem to imply very sparse occupancy of the postsynaptic membrane. This does not agree with what is known from quantitative ultrastructural studies [58–60] reviewed in [3] as well as others [51–57]. Furthermore, distribution skewness in this case is essentially the result of proximity to the lower limit on synaptic size, S = 0.

Collectively these findings indicate that unidirectional cooperativity in the form of the Contact Process, even with the addition of spontaneous binding to the matrix (α), fails to robustly account for the experimental observations described above, due to the inevitable tradeoff between distribution skewness and mean size.

### Bidirectional cooperativity model: Cooperative binding and unbinding

The inadequacy of unidirectional cooperative binding to capture the statistical features of synaptic populations led us to examine *bidirectional* cooperativity. In fact, although the Contact Process has been studied extensively, from a biophysical standpoint it is highly unlikely that binding is cooperative while unbinding is not.

**Fig 5:**
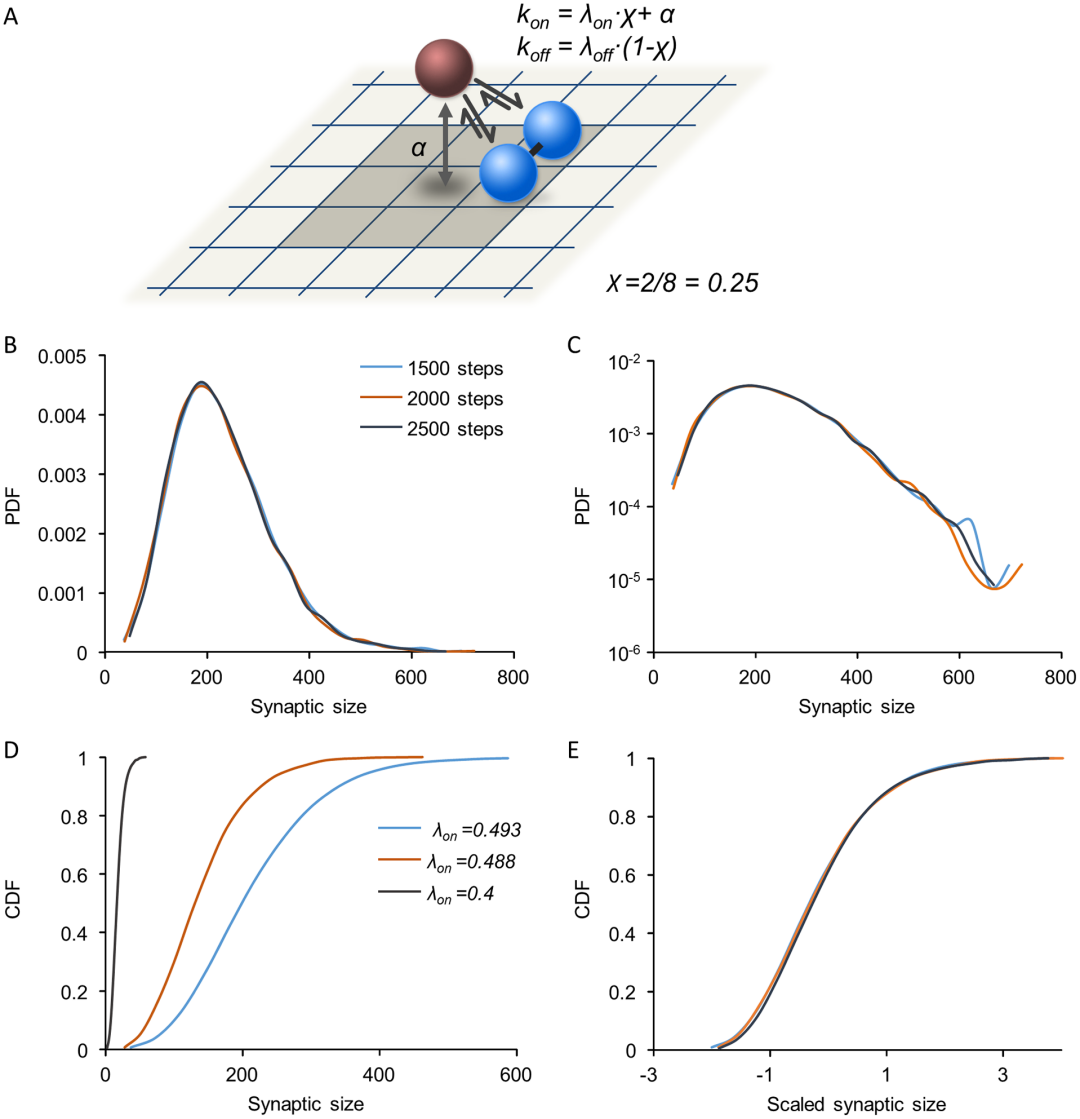
Synaptic size distributions in the bidirectional cooperativity model. **(A)** Illustration of the model: Both binding and unbinding are cooperative, with rates that depend on the fraction of occupied neighboring sites. Binding is also affected by a small, constant and non-cooperative component (*α*) representing weak, non-specific binding to the matrix. **(B)** Synaptic distributions are skewed and stable. Three distributions plotted at intervals of 500 simulation time steps. See Methods for simulation parameters. **(C)** Same distributions as in (B) plotted on a semi-logarithmic scale. **(D,E)** Size distributions at different parameter values show scaling: Cumulative distributions of synaptic sizes for different values of *λ*_*on*_ (D) in physical units, and the same distributions after scaling **(E)**.

In the bidirectional cooperativity model, the number of occupied neighboring sites modulates both binding and unbinding probabilities (Fig 5A). As the occupancy of neighboring sites increases, the probability of binding increases and the probability of unbinding decreases. Thus, the binding rate coefficient *k*_*on*_ and the unbinding rate coefficient *k*_*off*_ are expressed as follows:

$$k_{on}=\lambda_{on}\chi +\alpha \;and\;k_{off}=\lambda_{off}\left(1-\chi \right)$$ (4)

where *χ* is the fraction of occupied neighboring sites as before, and *λ*_*off*_ is the constant for cooperative unbinding (the maximal value of *k*_*off*_).

Stochastic simulations of the bidirectional cooperativity model reveal that, in contrast to the models described above, skewed synaptic size distributions can be obtained which are remarkably similar to those observed in experimental measurements (compare Fig 5B and 5C with Fig 2D). For a given parameter set, these model distributions are stable over time (Fig 5B). Moreover, synaptic size distributions in the model exhibit scaling similar to that observed experimentally (Fig 2E): Increasing the cooperative binding coefficient λ_on_ leads to a broadening of synaptic size distributions, which approximately collapse one on another after scaling (Fig 5D and 5E). Similar scaling is also observed when λ_off_ is modified.

**Fig 6:**
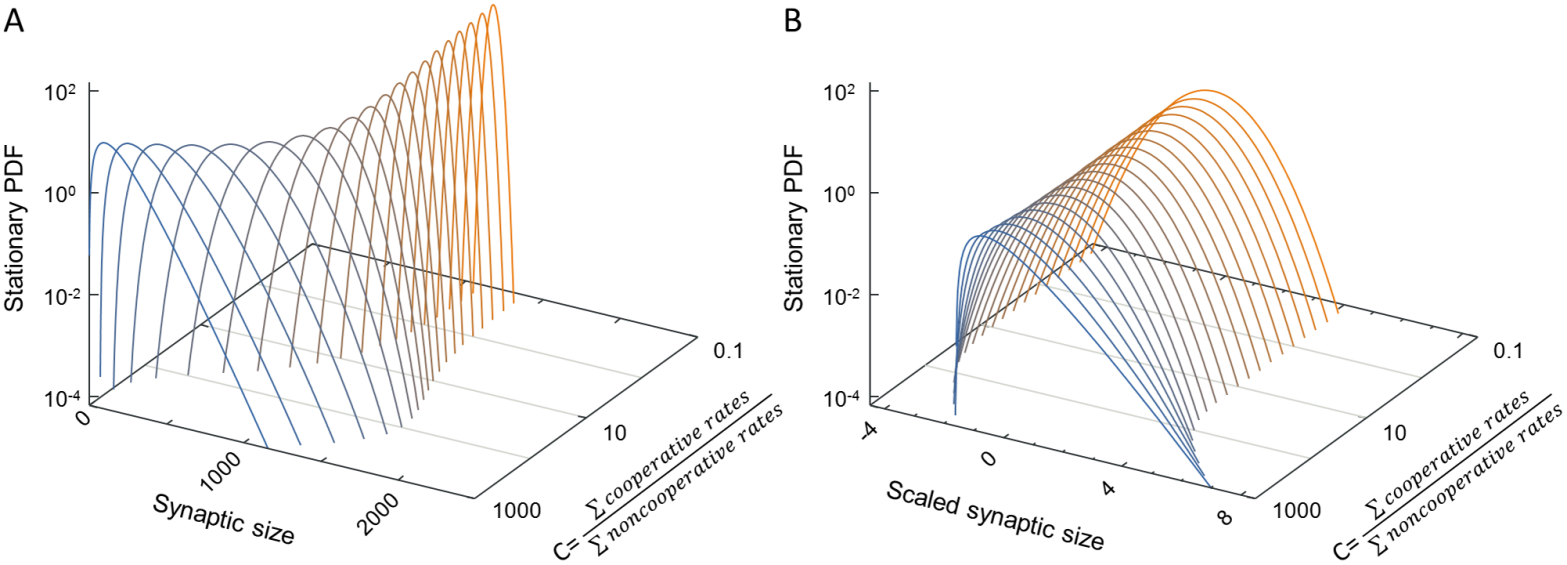
Stable stationary distributions in the global cooperativity model. Solutions of the global bidirectional cooperativity model, obtained by the Fokker-Planck approximation to the master equation (for details, see S1 Appendix, Section 2). (A) Distribution of synaptic sizes scanning a range of cooperativity parameter C (right-hand axis). For C<<1, we find stationary distributions very close to Gaussian (orange distributions). In this limit we approach a situation similar to the Langmuir model. For C>>1, when cooperative processes dominate, skewed distributions are found as observed experimentally (blue distributions). (B) Same stationary distributions as in (A), but scaled by subtracting the mean and dividing by the standard deviation. For technical reasons, a weak non-cooperative unbinding rate β was added (see S1 Appendix for a detailed justification); here β = α, and other parameters as in Methods.

We sought to identify the parameter regime that gives rise to such stable and skewed distributions in our model. Analysis of the continuum approximation of the model suggests that a stable distribution will be reached under the condition *α < λ*_*off*_*−λ*_*on*_ (see S1 Appendix, Section 1). To identify conditions for the emergence of skewness, we considered a simplified version of the bidirectional cooperativity model in which cooperativity acts globally on the entire synapse (see S1 Appendix, Section 2.1). This model still retains the main ingredient of cooperativity in both binding and unbinding processes but allows for solving the master equation, an equation for the probability for obtaining a certain occupancy state over time. The solution highlights a parameter combination that crucially affects the skewness of the steady-state distribution (see S1 Appendix, Sections 2.2,2.3). This is the cooperativity ratio C which characterizes the strength of cooperative binding and unbinding relative to the strength of the non-cooperative processes. In the model discussed here it is

$$C=\frac{\lambda_{on}+\lambda_{off}}{\alpha }$$ (5)

(In S1 Appendix, a more general version is considered of which this is a special case). Fig 6A shows the steady-state synaptic size distributions for the global cooperativity model over a wide range of this parameter. As intuitively expected, when C is small, binding and unbinding are dominated by non-cooperative processes and steady-state distributions are symmetric, narrow and Gaussian-like (orange distributions). When C is large, binding and unbinding are dominated by cooperative processes, and broad and skewed distributions can arise as observed in experiments (blue distributions). Fig 6B depicts the same distributions in scaled units, highlighting the skewness as a dimensionless shape characteristic. These results are congruent with simulations of the bidirectional cooperativity model where interactions between scaffold molecules are local (S1 Fig). To summarize, skewed distributions are obtained as long as cooperative binding is dominant relative to non-cooperative processes.

**Fig 7:**
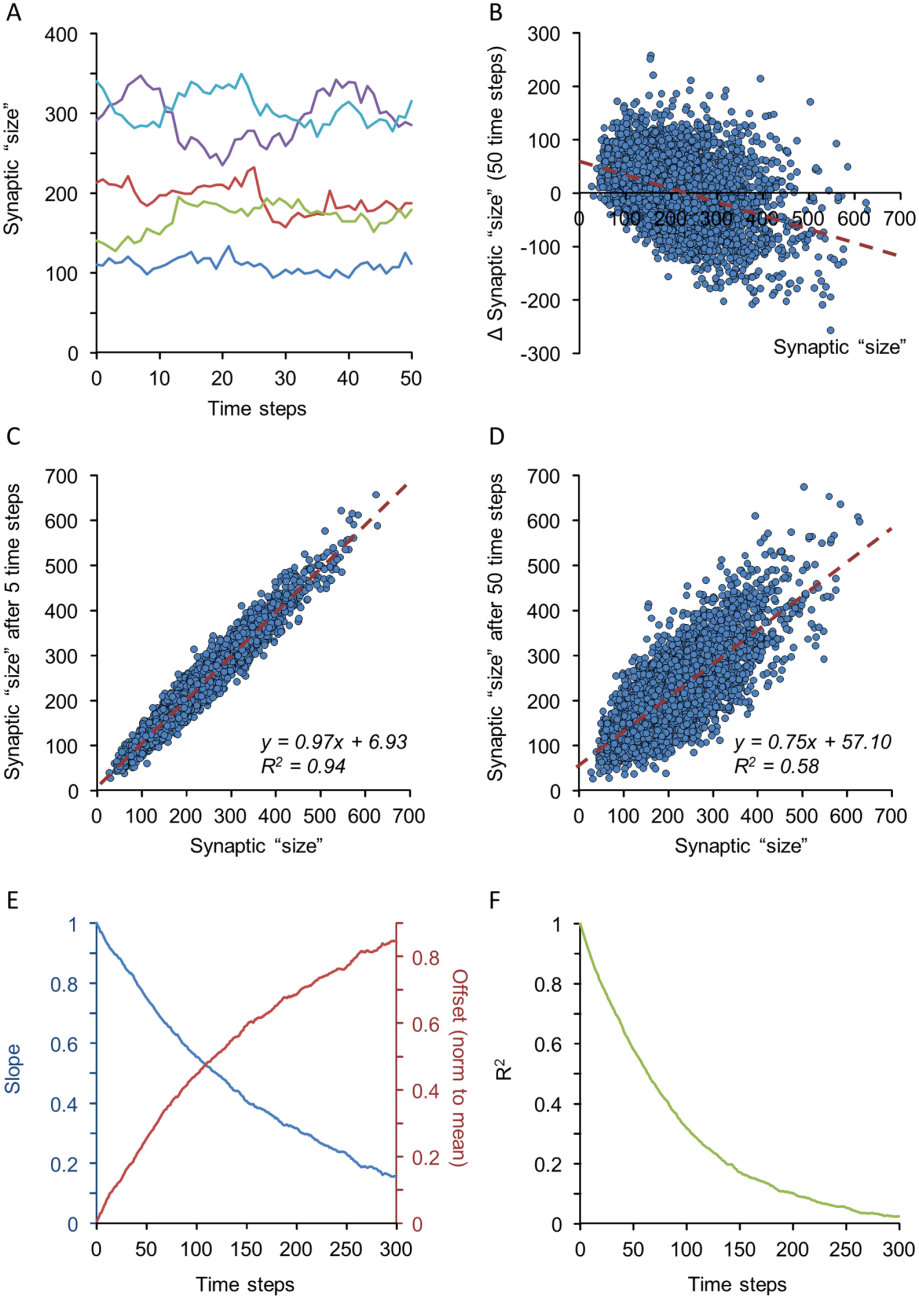
Synaptic size dynamics in the bidirectional cooperativity model. **(A)** Sizes of 5 simulated synapses over 50 simulation time steps. Note the fluctuations in simulated synapse size over the course of the simulation. **(B)** Scatter plot of changes in synapse size as a function of initial size after 50 time steps (3,500 synapses). **(C,D)** Changes over time of synaptic sizes for the same synapses after 5 (C) and 50 time-steps (D). Dashed red lines represent linear regression fits, with fit coefficients shown in the figure. **(E)** Slopes and offsets of linear regression lines in plots such as those of C and D for 300 consecutive time steps. Offsets were normalized to mean synaptic size during this 300 time-step window. **(F)** Coefficients of determination (R^2^) in plots such as those of C and D for 300 consecutive time steps. All data were obtained after mean synaptic size plateaued at ~225 bound molecules (after about 900 steps). See Methods for simulation parameters.

We examined whether the bidirectional cooperativity model also captures the dynamic properties of synaptic ensembles shown in Fig 2. Stochastic simulations show that sizes of individual synapses exhibit fluctuations that qualitatively resemble those observed for real synapses (Fig 7A; compare with Fig 2B). The scatter plot of changes in synaptic size as a function of their original size shows the same dependence observed experimentally (Fig 7B; compare with Fig 2C). Moreover, when plotting synaptic sizes as a function of their original sizes at increasingly greater time intervals (Fig 7C and 7D) the slopes and offsets of linear regression lines in such plots gradually decrease and increase respectively (Fig 7E), just as observed for excitatory [23] and inhibitory [19] synapses. Similarly, the coefficient of determination, or R^2^, gradually decreases (Fig 7F), suggesting a gradual “deterioration” of synaptic configurations as previously shown for excitatory and inhibitory [19] synapses.

### Spatial patterns of the bidirectional cooperativity model

**Fig 8:**
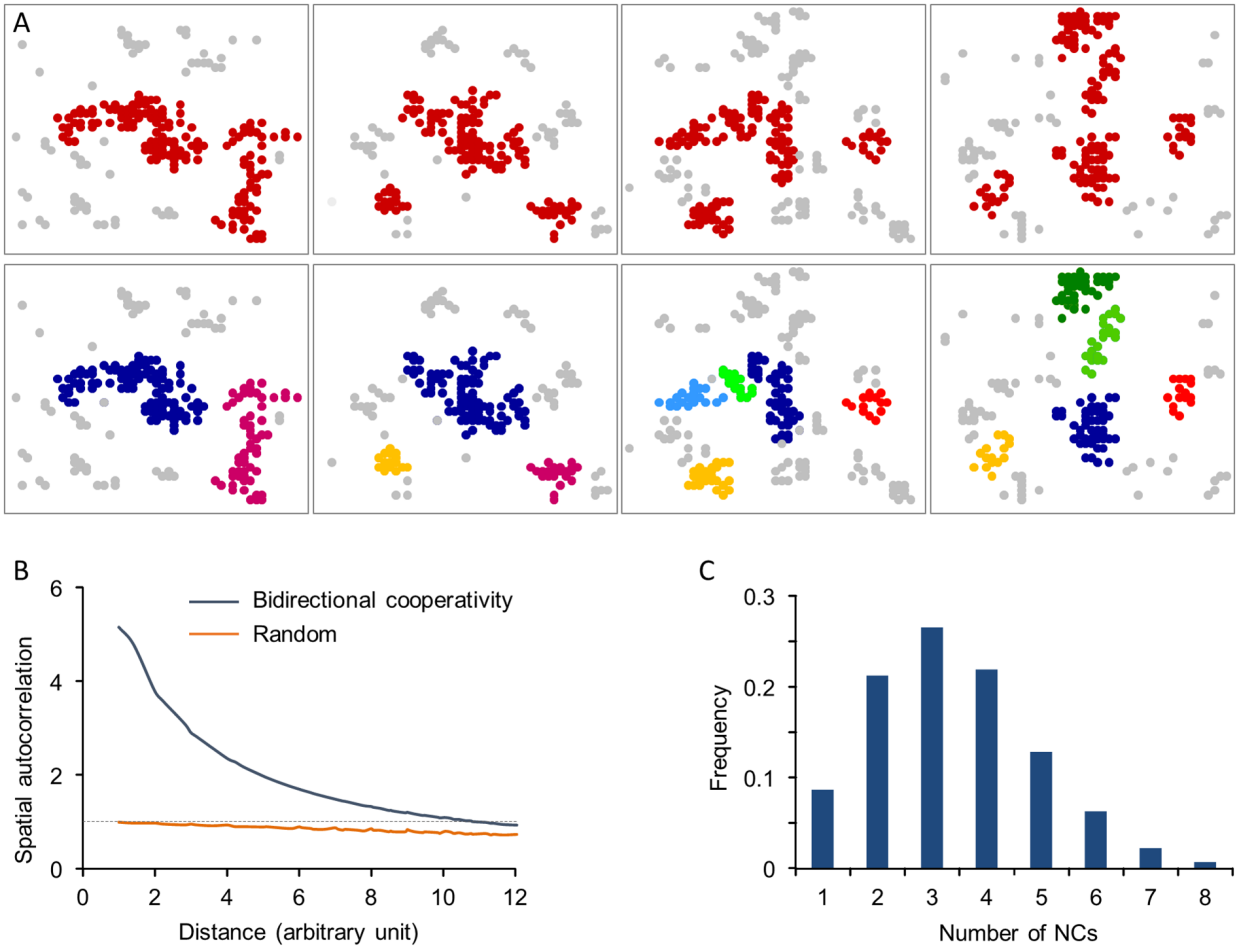
Spatial patterns of bound molecules and their evolution in the bidirectional cooperativity model. **(A)** “Time-lapse” images of molecules bound to the matrix. In the top panel, clusters are marked in red while sparse molecules in gray. In the bottom panel, clusters were separated using a hierarchical clustering algorithm (see Methods) with each cluster colored separately, allowing individual clusters to be followed over time. Time difference between consecutive frames is 10 steps. **(B)** Autocorrelation functions of synaptic structures obtained from the bidirectional cooperativity model and randomly distributed molecules. **(C)** Distribution of “nanocluster” numbers per synapse. See Methods for simulation details.

The findings described so far suggest that introducing bidirectional cooperativity allows the model to recapitulate the experimentally observed statistical properties of synaptic sizes in a population of synapses. In addition to these properties, recent experiments show that synapses, both inhibitory and excitatory, are not uniform structures but are organized as “nanoclusters” that change over time [51–57,72]. Does the same model recapitulate this dynamic internal organization of individual synapses? To test this, we examined the spatial patterns of bound molecules in our simulations and the changes in these patterns over time. We found that bound molecules do organize into nanocluster-like patterns (Fig 8A); moreover, “time-lapse” sequences revealed that these patterns “morph” in manners reminiscent of dynamics displayed by nanoclusters in glutamatergic synapses [51–53]. In fact, we note that spontaneous binding to the matrix (through the parameter α) creates transient “seeds” which can potentially nucleate nanocluster formation through cooperative binding to such seeds.

To further quantify this clustered organization, we used autocorrelation analyses (as in [56]) to characterize the degree of spatial heterogeneity in the simulated synaptic structures. The spatial autocorrelation function *g(r)* provides a measure of the molecular density at a distance *r* from a particular molecule relative to the average molecular density of the whole structure. Thus, when molecules are randomly distributed on the matrix, g(r) = 1. The extent to which g(r) exceeds 1 is related to the degree of clustering. As shown in Fig 8B, synaptic structures exhibit a much higher heterogeneity compared to randomly scattered molecules, indicating a high degree of clustering, in common with experimental observations [55,56].

We also quantified the number of nanoclusters formed in these simulations using the algorithm employed in [56] for identifying nanoclusters of synaptic scaffold proteins. As shown in Fig 8C, the average number of nanoclusters per synapse calculated by this method was 3.4±1.5 which is comparable to the number of PSD-95 nanoclusters observed experimentally (1.86 ± 0.07 per PSD) [56]. While the number of clusters in the model clearly depends on matrix size and model parameters, we note that these were initially chosen according to experimental observations as described above, giving roughly several hundred scaffold molecules per synapse. With the same parameters, the resulting numbers of clusters are also comparable with observed values.

## Discussion

In this study, we present a mesoscopic-level model, which provides an effective description of synaptic size dynamics. The basic premise of our approach is that the myriad microscopic processes that drive these dynamics can be effectively described as the net outcome of continuous, cooperative and stochastic assimilation and removal of synaptic (scaffold) proteins at a patch of postsynaptic membrane. We show that the introduction of cooperativity to both processes is indispensable and sufficient for generating distributions and dynamics qualitatively similar to those measured experimentally. Specifically, we show that the bidirectional cooperativity model captures the random-like changes in synaptic sizes, the non-Gaussian shape and long-term stability of synaptic size distributions, their scaling following various perturbations and the internal organization of synaptic molecules in nanoclusters. Our model thus points to the potentially fundamental role of cooperativity in dictating synaptic remodeling dynamics. Furthermore, our model offers a conceptually tractable understanding of synaptic remodeling dynamics, bridging the gap between non-intuitive, highly detailed molecular descriptions and abstract, low-dimensional statistical approaches. Although these conclusions were mainly based on Monte Carlo simulations, they are fully supported by analyses of the master equation for a simplified model of bidirectional global cooperativity (S1 Appendix).

### Limitations and robustness of the mesoscopic bidirectional cooperative model

The bidirectional cooperative model described here captures many features of synaptic dynamics previously observed in real neurons. Nevertheless, this mesoscopic model is undoubtedly simplistic and based on premises whose biological correctness is not obvious. Furthermore, its sensitivity to implementation details is not obvious either. Hence, matters of appropriateness and robustness warrant some discussion.

The first matter concerns the existence of a matrix as a binding substrate. At first sight, this would seem to be an entirely artefactual construct. The concept of a matrix, however, finds substantial justification when considering the fact that synapses form at contacts between pairs of elongated structures, that is axons and dendrites or dendritic spines; such contacts define and circumscribe regions within which axonal and dendritic molecules can interact across the synaptic cleft while simultaneously interacting with intracellular molecules such as scaffold molecules [73–75]. Thus, an axodendritic contact defines a specialized membrane patch that is effectively the equivalent of a matrix. The dimensions and geometry of such membrane patches undoubtedly vary, yet it is notable that our model produces broad and skewed distribution of synaptic sizes, even for uniform matrix sizes. Our results were not particularly sensitive to matrix size, as long as α was maintained at sufficiently low values such that non-cooperative binding did not become dominant, and only small numbers of “nanoclusters” were formed. For any given value of α, increased matrix size was associated with reduced skewness, which can be understood when considering that in these cases, synaptic size was the sum of sizes of many nanoclusters formed independently of each other, reducing the dominance of cooperativity and increasing the dominance of the independent binding. Interestingly, nanocluster numbers in real synapses tend to be very low [55,56] in agreement with this observation and its expected effects. As to other possible matrix geometries (such as hexagonal matrixes), alternatives were not explored; we did find, however, that smaller numbers of neighbors did not qualitatively affect our results (S2 Fig).

A second matter concerns the model’s simplicity—a single molecule type and only two types of interactions (Fig 5A). Real postsynaptic densities contain hundreds of different molecule types [76] which typically bind to multiple other molecules, creating a bewilderingly complex interaction network [2,30,76]. The dynamics arising from such rich and complex collections of interacting molecules remain unknown, yet we tentatively suggest that the principles we outline here may hold in general: the more molecules bound to the postsynaptic matrix, the higher the probability of recruiting additional molecules to the same matrix. Conversely, the greater the number of molecules a particular molecule is bound to, the lower its probability of dissociating from the matrix. Indeed, *in-vivo* measurements of PSD-95 molecular dynamics [9] suggest that large PSDs capture more free PSD-95 and retain it for longer durations as compared to small PSDs. We thus expect that this form of cooperativity (sometimes referred to as avidity [64]) will give rise to qualitatively similar dynamics and population properties.

A third matter concerns the linear dependence of binding and unbinding rates on the number of bound neighbors. The exact description of binding kinetics and their relation to physical interactions is a highly nontrivial aspect of surface science, even for relatively simple physical interactions [77]. Energy considerations and detailed balance impose some constraints but do not define the kinetics uniquely. All the more in our model, which is highly abstract and provides no more than a simplified sketch of a synaptic molecule assembly. We used linear dependence since it provided a simple realization of the principle of cooperativity as described above. Moreover, it enabled us to analyze a corresponding global cooperativity model, and obtain solutions of its master equation (S1 Appendix). Nevertheless, we cannot exclude the possibility that our findings may not apply universally to all possible cooperativity models.

A final matter concerns the parameter regimes used here. We noted that this regime is constrained by several considerations. We found that it is important to keep non-cooperative binding rates much smaller than cooperative rates in order to obtain skewed distributions of synaptic sizes; this is in line with the large number of interaction partners most synaptic molecules have. Additionally, values of λ_on_ very close to those of λ_off_ were required in order to obtain “reasonable” mean synaptic sizes (in terms of matrix occupancy; S3 Fig). At first sight, this requirement would seem to question the model’s robustness. We note, however, that from a biophysical standpoint, λ_on_ encompasses not only particular binding kinetics but also the concentration of free molecules that can potentially bind to the matrix; put differently, the rates at which molecules bind to the matrix are also proportional to free molecule concentrations. In our treatment so far, this dependence was not made explicit, and free molecule concentration was encapsulated in λ_on_. Separating λ_on_ into these two components, however, gives rise to an interesting observation (S4A Fig): For a broad range of total molecule concentrations, λ_on_ settles on values that are very close to those of λ_off_. Consequently, even when total molecule concentrations are changed several fold, the condition λ_on_≈λ_off_ is maintained and distributions of synaptic sizes remain skewed and stable. These same changes, however, affect mean synaptic size dramatically, (S4B Fig). In summary, changing total molecule concentrations (readily realized by altering protein synthesis or degradation rates, for example) changes mean synaptic sizes and drives synaptic size distribution scaling (as previously suggested, e.g. [48,50]), yet only minimally affects λ_on_, which remains very close to λ_off_. Consequently, the parametric regime λ_on_≈λ_off_ is very reasonable in the context of our model.

### Significance to synaptic remodeling dynamics and population properties

Mesoscopic models in which the synapse is described as an assembly of dynamic molecules have been put forward in several prior studies. Thus, for example, Shouval [22] in an approach already mentioned, depicted the synapse as a matrix to which neurotransmitter receptors can be added or removed. In a second study [21] the synapse was modeled as a three layer system divided laterally into synaptic and extrasynaptic regions. It was shown that cooperative interactions between synaptic molecules could give rise to persistent postsynaptic sites, which transiently trap receptors as they diffuse laterally in the plasma membrane. In a third approach [11], a model based on reaction-diffusion equations for scaffold proteins and receptors was shown to give rise to postsynaptic domains (via a Turing mechanism), that coexist with rapid receptor diffusion in the cell membrane plane. All these studies were aimed at explaining the long-term persistence of synapses in face of continuous diffusion, exchange and turnover of their molecular constituents. Very recently, a mesoscopic biophysical model based on diffusion, aggregation and removal of receptors and scaffold proteins in the membrane was used to explain the statistics of PSD molecule clusters [72]. None of these models, however, examined how such molecular dynamics may give rise to spontaneous synaptic remodeling or population properties such as size distribution shapes or their scaling. Conversely, the mesoscopic model described here shows how these properties emerge naturally from simple well-known biological processes, namely cooperative binding and unbinding, and by doing so provides a conceptually tractable explanation of these phenomena. Clearly, as mentioned above, it is an enormously simplified description of the postsynaptic specialization. However, its main ingredients—a postsynaptic membrane, dynamic molecules that continuously bind and unbind, and a strong tendency of such molecules to interact with multiple other molecules—are now well established facts. We thus carefully suggest that the nanoscale organization of synaptic scaffolds, the spontaneous, size dependent fluctuations in synaptic sizes, the gradual erosion of synaptic configurations, the skewed distribution of synaptic sizes and their scaling in response to global changes in synaptic molecule concentrations, are all likely to be driven, at least in part, by spontaneously occurring cooperative assimilation and loss of synaptic molecules. Naturally, real synapses will have many additional means of control through which they might change specific binding and unbinding affinities, the repertoire and abundance of synaptic molecules and the supply of metabolic energy required to fuel some of these reactions. Nevertheless, we conjecture that these additional means are layered upon foundations consisting of principles exposed by our simplistic model.

### Cooperativity as an organizing principle

Cooperativity is a ubiquitous and crucial regulation mechanism in a large variety of processes, including molecular recognition, enzyme catalysis, membrane transport, protein folding, and self-assembly of supramolecular complexes [63–69]. In the context of synaptic biology, cooperativity plays key roles not only in the formation of multi-molecular scaffolds (e.g. [78–83]) but also in synaptic function, where it is mostly appreciated in relation to neurotransmitter release [84]. Along these lines it is intriguing to note the considerable functional variability in space and time exhibited by presynaptic boutons as well as the skewed shape of various presynaptic property distributions (e.g. [85–89]; reviewed in [90]). Interestingly, skewed distributions [91,92] as well as nonstationary properties (e.g. [93,94]; reviewed in [95]) feature prominently in neuronal functional and structural features.

As a final note we wish to remark that our model, in its most generic and abstract sense, concerns the dynamics and statistical outcomes of stochastic, cooperative construction and deconstruction processes; consequently, the study’s conclusions are not necessarily limited to synaptic, neuronal, or, for that matter, biological settings. In fact, it is reasonable to expect that when collections of multiple instantiations of cooperative constructive and deconstructive processes are examined, these might exhibit features similar to those described here, that is, state dependent fluctuations in the properties of individual instantiations, and, at the same time, skewed and stable distributions of the same properties in populations of such instantiations.

## Methods

### Simulations

All simulations were performed using scripts written in Matlab (MathWorks, MA, USA). A number of simulations were also repeated using code written in C. Monte Carlo simulations were performed to assess the dynamics and statistics that result from each one of the three models and test their congruence with experimental measurements. Specifically, for each model, the trajectories of 3500 synapses were simulated over 1500 time steps. At each time step and for each site, the fraction of occupied nearest neighbors χ was calculated by counting the number of occupied nearest neighbors and dividing it by the total number of nearest neighbors. The binding and unbinding probabilities for vacant and occupied sites, respectively, were determined by the mode of interactions presented by each model. A site changed its binding state if the probability calculated for this site was larger than a random number sampled from a uniform distribution between 0 and 1.

Unless stated otherwise, we used the following parameter values: *λ*_*off*_ = 0.5 *t*^−1^, *λ*_*on*_ = 0.493 *t*^−1^, *α* = 0.0007 *t*^−1^ (t stands for time). The geometry of the postsynaptic density was chosen, for reasons of simplicity, to be a 50x50 square matrix, giving a total of M = 2,500 sites. In this geometry, the maximal number of nearest neighbors is 8 and the fraction of occupied nearest neighbors χ was calculated accordingly. Parameters were chosen to give roughly several hundred scaffold proteins per synapse as observed for glutamatergic synapses in the mammalian central nervous systems [3].

Simulations were performed using a time step of 1 (arbitrary units). Results were not significantly altered when time steps were decreased by factors of 2 to 100.

Code used for all MATLAB simulations is provided as S1 Code.

### Spatial autocorrelation analysis

Spatial autocorrelation analysis was used to quantify the clustering of synaptic proteins. The autocorrelation function *g*(*r*) is a measure of bound protein density at a distance r away from a given bound protein relative to the density of the whole matrix. The density at a certain distance r was calculated by averaging the number of occupied sites at distance r from each occupied site and dividing it by the total number of sites at distance r. The autocorrelation function was then obtained by performing the same calculation for different values of r and normalizing it by the density of the whole matrix. The case r = 0 was not considered due to its trivial contribution. For a higher precision, this analysis was performed for 3500 synapses and the autocorrelation function was taken as their average.

### Cluster analysis

The number of nanoclusters in the bidirectional cooperativity model was calculated using agglomerative hierarchical clustering algorithm as employed in [56] for analyzing scaffold proteins nanoclusters. Occupied sites were partitioned into sub-clusters using MATLAB functions pdist(), linkage() and cluster(). The node height cut-off of the dendrogram was determined by the mean of nearest neighbor distances between occupied sites + 2 standard deviations. This analysis was performed for each time point to measure the morphing of clusters in time.

## Supporting information

S1 Fig Dependence of synaptic size distribution skewness on α in the bidirectional cooperativity model. Simulations were run using increasing values of α. At the end of each simulation, the distribution of synaptic sizes was calculated for the last time point and its skewness was computed. Values of all other parameters were kept constant and set to the values listed in Methods. Note the sharp decrease in skewness as α becomes greater. Averages and standard deviations of 5 repeats.
(PDF)

S2 Fig Synaptic size distributions obtained when considering four or eight nearest neighbors. **(A)** Illustration of the bidirectional cooperativity model in which only four nearest neighbors (shaded) are considered for the calculation of χ. **(B)** Synaptic size distributions for simulations considering four and eight nearest neighbors. Skewed distributions are obtained for both cases. **(C)** Scaled versions of the distributions shown in B) show that their shapes are very similar.
(PDF)

S3 Fig Dependence of synaptic size average and size distribution skewness on λ_on_. Simulations of the bidirectional cooperativity model were performed for different values of λ_on_ while holding values of all other parameters, and in particular, λ_off_, fixed to values mentioned in Methods. **(A)** Mean synaptic size is dramatically smaller for values of λ_on_ that are very far from λ_off_. This is consistent with relationships between mean synaptic size and λ_on_ resolved analytically in the mean-field treatment (S1 Appendix). **(B)** The skewness is not sensitive to the value of λ_on_ until its value becomes very close to λ_off_. The decrease of skewness in this case stems from the finite size effect of the matrix that becomes more significant for larger means. Averages and standard deviations of 10 repeats.
(PDF)

S4 Fig The condition λ_on_≈λ_off_ is maintained over a large range of total molecule concentrations. **(A)** To examine how λ_on_ is affected by changes in cellular molecule concentration, simulations were performed as described in the main text except that here, it was assumed that all synapses belong to the same cell and share a common pool of molecules. In addition, the dependence of binding rates on free molecule concentration was made explicit such that at every time point, λ_on_effective_ = *N*_*free*_·λ*_on_, with *N*_*free*_ representing the momentary concentration of free molecules. Consequently, in these simulations *k*_*on*_ *= N*_*free*_ · (λ*_on_ *· χ + α)*. At each step of the simulation, *N*_*free*_ was updated by subtracting the numbers of molecules bound to all synapses from the predefined number of total molecules such that *N*_*free*_ = *N*_*total*_*−N*_*bound*_. This simulation was run for 1,500 steps for 1,000 synapses; in each run *N*_*total*_ was set to a different value whereas λ*_on_, *λ*_*off*_ and *α* were kept the same (5*·*10^−6^, 0.5 and 0.0007 respectively, as in Figs 3–5, 7 and 8). At the end of each simulation, λ_on_effective_ was calculated based on *N*_*free*_ and its values for the last 10 simulation steps were averaged. Average *λ*_*on_effective*_ was then plotted against *N*_*total*_. Note that >4-fold changes in *N*_*total*_ barely affected *λ*_*on*_ which settled on values very close to those of *λ*_*off*_. **(B)** Mean synaptic size in the same simulations as a function of *N*_*total*_. Note the nearly linear increase in mean synaptic size with increasing values of *N*_*total*_.
(PDF)

S1 Appendix This appendix contains analysis of several binding and unbinding models using a master equation approach. In particular the bidirectional cooperativity model with global cooperativity, found to display similar statistical properties to the local cooperativity model describe in the main text, is analyzed. Formulas are developed for the steady-state distribution in the Fokker-Planck approximation for this case, which allow the efficient scanning of parameter space and the identification of parameters relevant for the skewness of the distribution.
(PDF)

S1 Code Source code (Matlab) used for simulations. (ZIP)
