# Supplementary figures and images for: Cooperative stochastic binding and unbinding explain synaptic size dynamics and statistics
Source: PLoS Comput Biol. 2017 Jul 13;13(7):e1005668. doi: 10.1371/journal.pcbi.1005668 (PMC5546711; doi:10.1371/journal.pcbi.1005668)

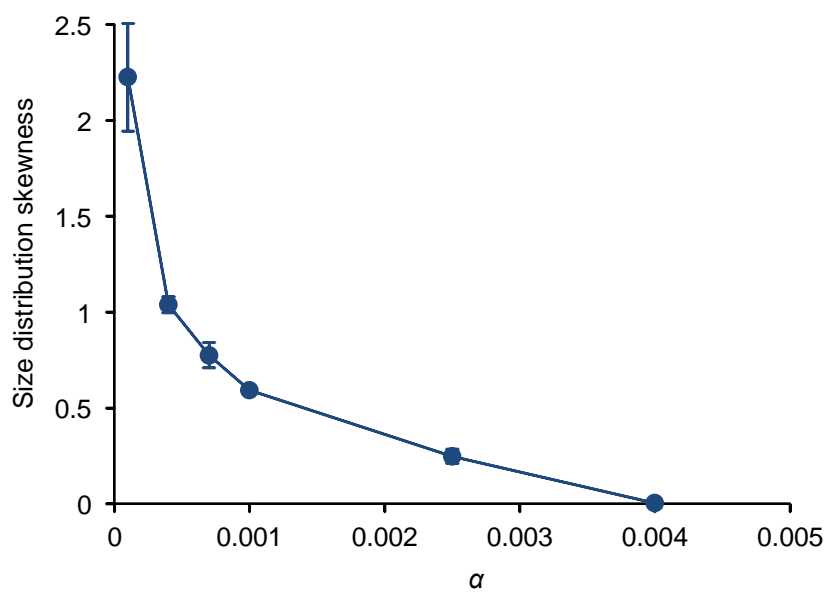

Figure S1  
Shomar et al.

Supplement: S1 Fig — Simulations were run using increasing values of α. At the end of each simulation, the distribution of synaptic sizes was calculated for the last time point and its skewness was computed. Values of all other parameters were kept constant and set to the values listed in Methods. Note the sharp decrease in skewness as α becomes greater. Averages and standard deviations of 5 repeats. (PDF) [file pcbi.1005668.s001.pdf]

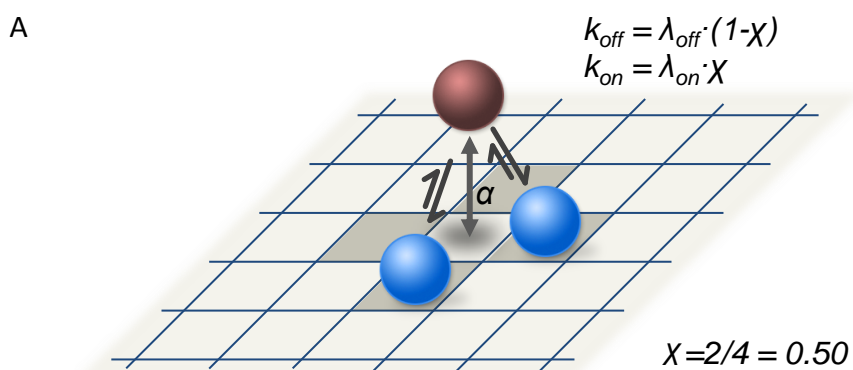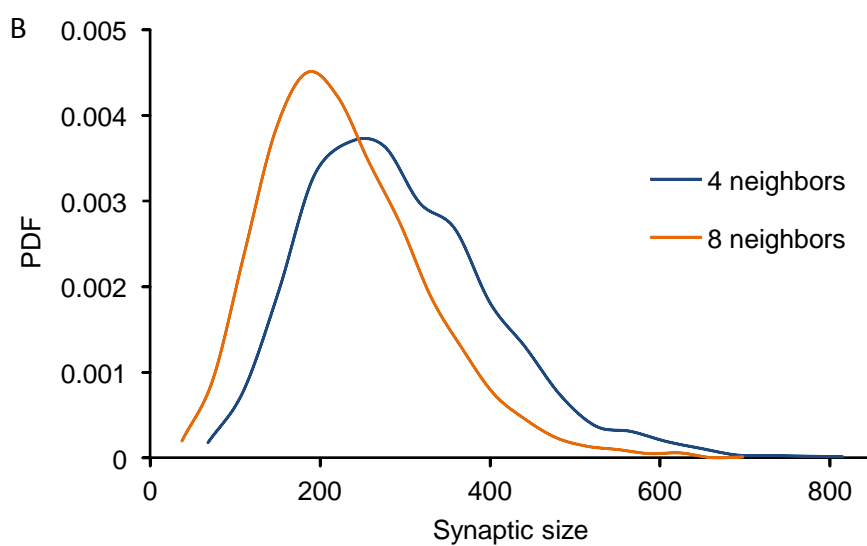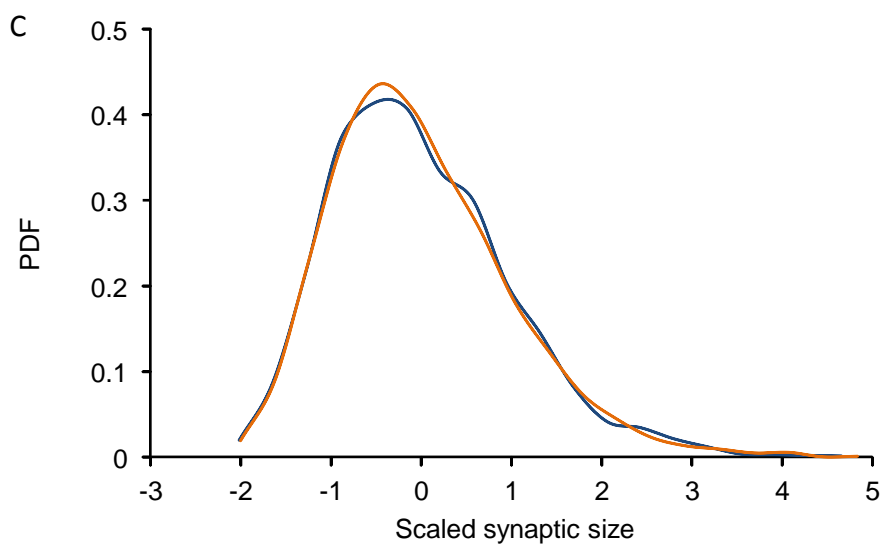

Figure S2  
Shomar et al.

Supplement: S2 Fig — (A) Illustration of the bidirectional cooperativity model in which only four nearest neighbors (shaded) are considered for the calculation of χ. (B) Synaptic size distributions for simulations considering four and eight nearest neighbors. Skewed distributions are obtained for both cases. (C) Scaled versions of the distributions shown in B) show that their shapes are very similar. (PDF) [file pcbi.1005668.s002.pdf]

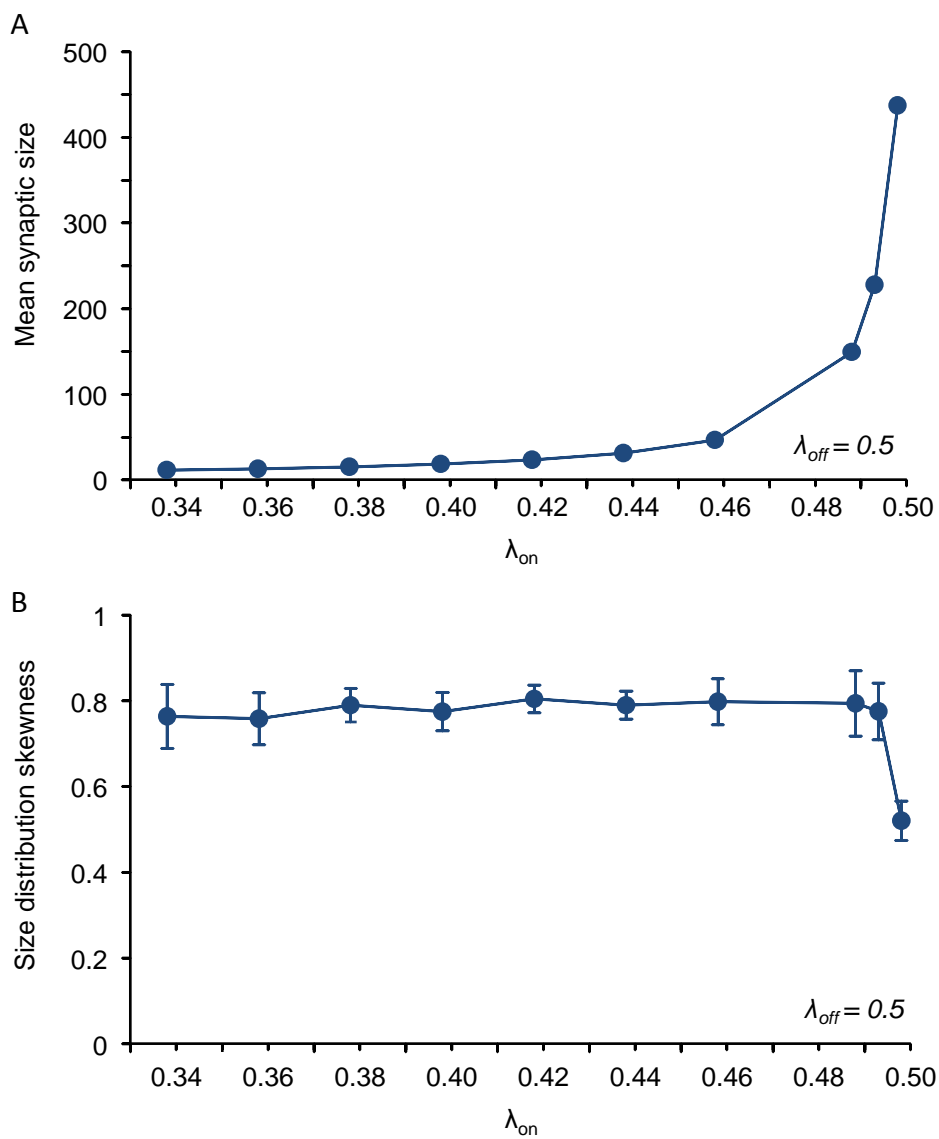

Figure S3  
Shomar et al.

Supplement: S3 Fig — Simulations of the bidirectional cooperativity model were performed for different values of λon while holding values of all other parameters, and in particular, λoff, fixed to values mentioned in Methods. (A) Mean synaptic size is dramatically smaller for values of λon that are very far from λoff. This is consistent with relationships between mean synaptic size and λon resolved analytically in the mean-field treatment (S1 Appendix). (B) The skewness is not sensitive to the value of λon until its value becomes very close to λoff. The decrease of skewness in this case stems from the finite size effect of the matrix that becomes more significant for larger means. Averages and standard deviations of 10 repeats. (PDF) [file pcbi.1005668.s003.pdf]

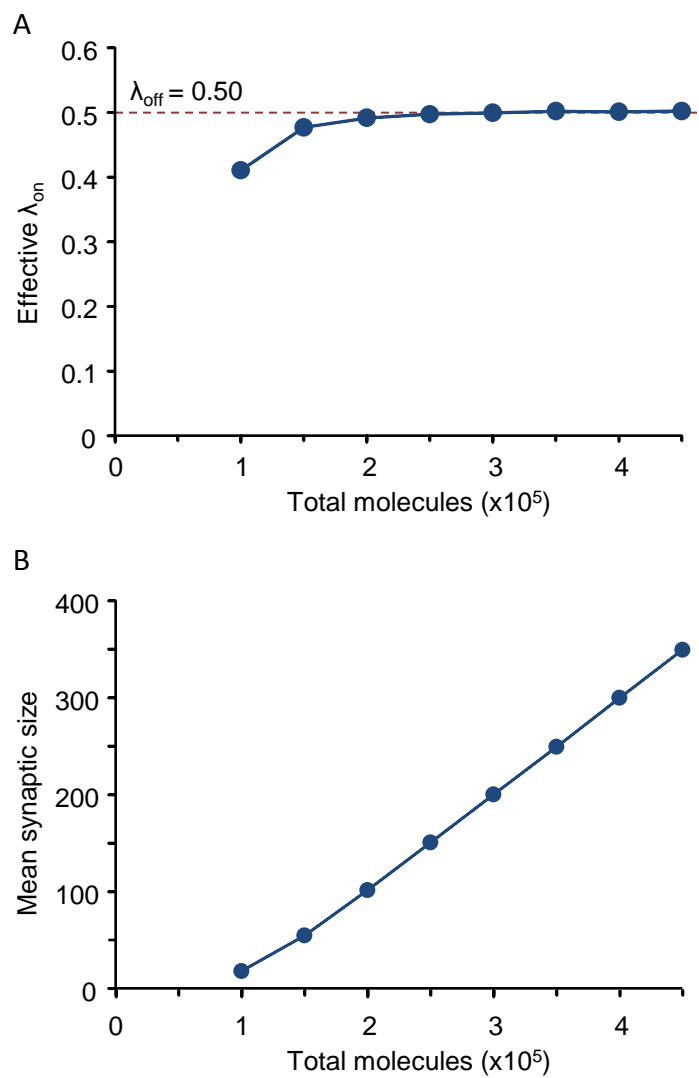

Figure S4  
Shomar et al.

Supplement: S4 Fig — (A) To examine how λon is affected by changes in cellular molecule concentration, simulations were performed as described in the main text except that here, it was assumed that all synapses belong to the same cell and share a common pool of molecules. In addition, the dependence of binding rates on free molecule concentration was made explicit such that at every time point, λon_effective = Nfree·λ*on, with Nfree representing the momentary concentration of free molecules. Consequently, in these simulations kon = Nfree · (λ*on · χ + α). At each step of the simulation, Nfree was updated by subtracting the numbers of molecules bound to all synapses from the predefined number of total molecules such that Nfree = Ntotal−Nbound. This simulation was run for 1,500 steps for 1,000 synapses; in each run Ntotal was set to a different value whereas λ*on, λoff and α were kept the same (5·10−6, 0.5 and 0.0007 respectively, as in Figs 3–5, 7 and 8). At the end of each simulation, λon_effective was calculated based on Nfree and its values for the last 10 simulation steps were averaged. Average λon_effective was then plotted against Ntotal. Note that >4-fold changes in Ntotal barely affected λon which settled on values very close to those of λoff. (B) Mean synaptic size in the same simulations as a function of Ntotal. Note the nearly linear increase in mean synaptic size with increasing values of Ntotal. (PDF) [file pcbi.1005668.s004.pdf]
