# Supplementary material for: Cooperative stochastic binding and unbinding explain synaptic size dynamics and statistics
Source: PLoS Comput Biol. 2017 Jul 13;13(7):e1005668. doi: 10.1371/journal.pcbi.1005668 (PMC5546711; doi:10.1371/journal.pcbi.1005668)
Supplement: S1 Appendix — In particular the bidirectional cooperativity model with global cooperativity, found to display similar statistical properties to the local cooperativity model describe in the main text, is analyzed. Formulas are developed for the steady-state distribution in the Fokker-Planck approximation for this case, which allow the efficient scanning of parameter space and the identification of parameters relevant for the skewness of the distribution. (PDF) [file pcbi.1005668.s005.pdf]

S1 Appendix: Cooperative Stochastic Binding and Unbinding Explain Synaptic Size Dynamics and Statistics

Aseel Shomar, Lukas Geyrhofer, Noam E. Ziv, Naama Brenner

1 Stability analysis for bidirectional cooperativity: mean-field approximation

In order to determine the appropriate parameter regime for the bidirectional cooperative model, we first analyze its rate equations in the mean-field approximation. This approximation neglects fluctuations altogether, but can provide some information on qualitative behavior on e.g. stability and the existence of phase transitions. We define $\Theta = S/M$ to be the fraction of occupied sites on the lattice; with this notation, the fraction obeys the average equation

$$\begin{aligned} \partial_t \Theta &= k_{\text{on}}(1-\Theta) - k_{\text{off}}\Theta \\ &= \big((\lambda_{\text{on}} - \lambda_{\text{off}})\Theta + \alpha\big)(1-\Theta)\ . \end{aligned} \tag{S1}$$

Eq. (S1) has two stationary states, $\Theta^* = 1$ and $\Theta^* = -\alpha/(\lambda_{\text{on}} - \lambda_{\text{off}})$. While the full matrix ($\Theta^* = 1$) state is always a solution of this equation, some parameters combinations render the latter fixed point outside the admissible interval between 0 and 1. A linear stability analysis reveals that actually only one of these fixed points is stable – they switch their respective stabilities when the expression $-\alpha/(\lambda_{\text{on}} - \lambda_{\text{off}})$ enters the interval $\big[0;1\big]$ (see Fig. SI1).

2 A stochastic model with global cooperativity

In the full stochastic bidirectional cooperativity model (probabilities defined by Eq. 4 in the main text), binding and unbinding depends on the number of occupied nearest-neighbor lattice sites. This is biophysically plausible since inter-molecular interactions are of limited range. A mathematically simpler model can be

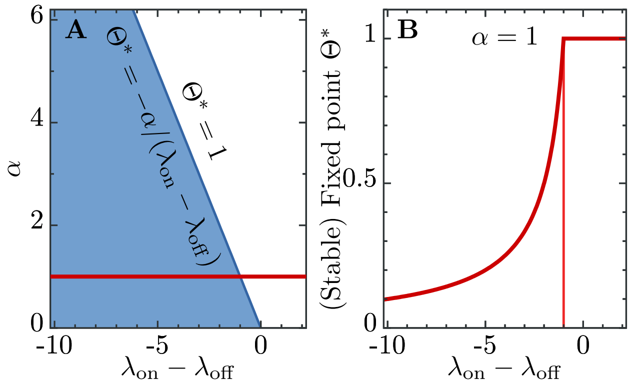

Figure SI1: **Stability of a finite-fraction occupancy in the mean-field approximation.** (A) Region of parameter space $(\alpha, \lambda_{\text{on}} - \lambda_{\text{off}})$ with corresponding stable fixed points. In each region only one stable fixed point exists, either full coverage $\Theta^* = 1$ or an intermediate fraction. (B) Value of the stable fixed point as a function of $\lambda_{\text{on}} - \lambda_{\text{off}}$.

defined where binding and unbinding are stochastic events, but their probabilities exhibit a global bidirectional cooperativity: they depend on the entire occupancy of the lattice, regardless of where the occupied sites are located:

$$k_{\rm on} = \lambda_{\rm on}\Theta + \alpha \ , \qquad \text{and} \quad k_{\rm off} = \lambda_{\rm off}(1-\Theta) \ . \tag{S2}$$

Below we present numerical simulations of this model and analysis of its Master equation for the probability distribution of synaptic size.

2.1 Statistical properties of the global cooperativity model

Simulations of this global interaction model reveal that it displays statistical properties similar to the local bidirectional model. Fig. SI2 shows the analog of Fig. 4 in the main text, demonstrating the shape, stability and scaling properties of synaptic size distributions in the global interaction model. Fig. SI3A-D displays properties of the temporal fluctuations in synaptic size in this model, in analogy to Fig. 5 in the main text. The temporal properties are similar. However, the spatial patterns consisting of clusters shown in Fig. 6 of the main text are completely absent, as clearly expected, since interactions are homogeneous throughout the lattice (Fig. SI3E).

The similarity of distribution shape and scaling properties between the global and local cooperativity model motivated us to investigate the Master Equation for synaptic size in the global model in more detail. Since all probabilities are determined by one variable - total synaptic size - the equation is relatively simple and can be analyzed to help understand the origin of skewed distributions in the presence of bidirectional cooperativity.

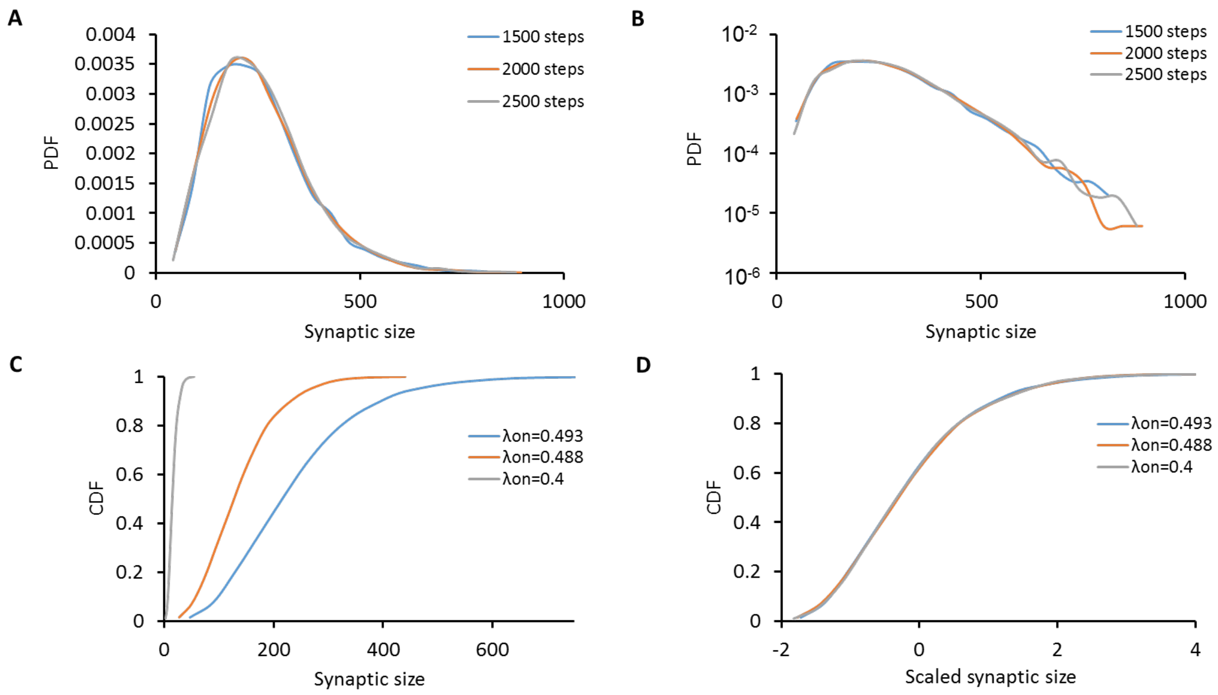

Figure SI2: **Statistical properties of the bidirectional cooperativity model with global interactions over the entire lattice.** Monte Carlo simulations were performed on a lattice with probabilities of binding and unbinding depending on the entire lattice occupancy. (A) Synaptic distributions are skewed and stable. Three distributions plotted at intervals of 500 simulation time steps. See Methods for simulation parameters. (B) Same distributions as in (A) plotted on a semi-logarithmic scale. (C) Cumulative distributions of synaptic sizes for different values of $\lambda_{\rm on}$. (D) Same distributions as shown in (C) after scaling. Size distributions at different parameter values collapse after scaling.

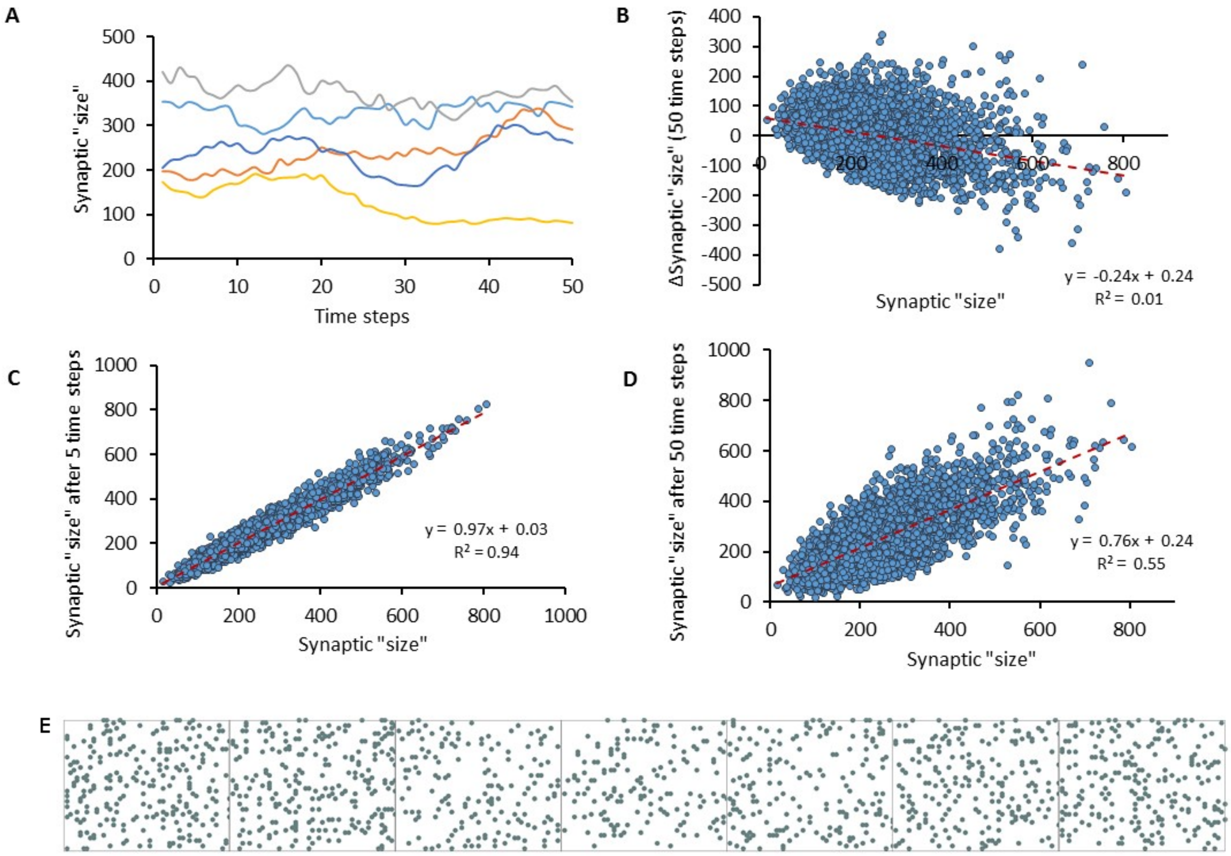

Figure SI3: **Synaptic size dynamics in the bidirectional cooperativity model with global interactions over the entire lattice.** (A) Sizes of 5 simulated synapses over 50 time steps. Note the fluctuations in synapse size over the course of the simulation. (B) Scatter plot of changes in synapse size as a function of initial size after 50 time steps (3500 synapses). (C,D) Mapping between momentary synaptic size and its value after 5 (C) and 50 time-steps (D). Dashed red lines represent linear regression fits, with fit coefficients shown in the figure. (E) Spatial patterns of bound molecules and their evolution. "Time-lapse" images of molecules bound to the matrix. Time difference between consecutive frames is 10 steps. Note that bound molecules are not organized in clusters. See Methods for simulation parameters.

2.2 Master Equation for global models - general

In order to analyze the distribution of synaptic sizes, we formulate the master equation for stochastic binding and unbinding of molecules to the membrane matrix, where cooperativity may be introduced globally. We formulate this as a general model with different parameter sets corresponding to the Langmuir, Contact or global bidirectional cooperativity model. All these models share the property that binding and unbinding can (at most) depend on the total binding state of the synapse and not on its spatial configuration. In the following, we introduce the abbreviations $A(\Theta) = k_{\text{on}}(1-\Theta)$ and $B(\Theta) = k_{\text{off}}\Theta$ for the total binding and unbinding fluxes per unit time, respectively. This notation is general for all models discussed in the main text. These models differ in their dependence of binding/unbinding probabilities on the fraction of bound synaptic molecules; specifically,

$$\begin{aligned}
\text{Langmuir model} \quad & k_{\text{on}} = \alpha\ , & k_{\text{off}} &= \beta\ , \\
\text{Contact process} \quad & k_{\text{on}} = \lambda_{\text{on}}\Theta + \alpha\ , & k_{\text{off}} &= \beta\ , \\
\text{Bidirectional cooperative} \quad & k_{\text{on}} = \lambda_{\text{on}}\Theta + \alpha\ , & k_{\text{off}} &= \lambda_{\text{off}}(1-\Theta)\ .
\end{aligned} \tag{S3}$$

In the most general case a non-cooperative unbinding rate β can be added also to the bidirectional cooperativity model. These in turn define the specific functions $A(\Theta)$ and $B(\Theta)$ for each model.

In a discrete master equation for a population of synapses, one only considers changes of the system size

S by binding or unbinding of a single molecule; the time evolution of the probability $\mathbb{P}[\Theta,t]$ is then

$$\partial_t \mathbb{P}[\Theta,t] = A(\Theta-\tfrac{1}{M})\mathbb{P}[\Theta-\tfrac{1}{M},t] + B(\Theta+\tfrac{1}{M})\mathbb{P}[\Theta+\tfrac{1}{M},t] - A(\Theta)\mathbb{P}[\Theta,t] - B(\Theta)\mathbb{P}[\Theta,t] \ . \tag{S4}$$

In principle, the probabilities $\mathbb{P}[\Theta,t]$ are only defined on a the discrete set of values of Θ with distance $1/M$ to each other. For system size M large enough, however, we can assume Θ to be (almost) continuous. This allows to approximate the discrete coupling to neighboring probabilities in Eq. (S4) via series expansions in orders of $1/M$. Zeroth-order terms of this expansion, $A(\Theta)\mathbb{P}[\Theta,t]$ and $B(\Theta)\mathbb{P}[\Theta,t]$, cancel with their counter-terms in the original master equation (S4). After keeping only terms up to second order in $1/M$, we arrive at a Fokker-Planck equation,

$$\partial_t \mathbb{P}[\Theta,t] = -\partial_\Theta \left[\frac{A(\Theta)-B(\Theta)}{M}\mathbb{P}[\Theta,t]\right] + \partial_\Theta^2 \left[\frac{A(\Theta)+B(\Theta)}{2M^2}\mathbb{P}[\Theta,t]\right] \ , \tag{S5}$$

as an approximation to the original master equation, Eq. (S4). The stationary distribution, $\partial_t \mathbb{P}_\star[\Theta]=0$, to Eq. (S5) is given by the expression

$$\log \mathbb{P}_\star = 2M \int_\Theta \frac{A-B}{A+B} - \log(A+B) + c' \ , \tag{S6}$$

where the constant c' ensures normalization of the stationary distribution, $\int_0^1 d\Theta \ \mathbb{P}_\star[\Theta] = 1$.

Similar to the introduction of the non-cooperative binding rate α, that prevents the dynamical system collapsing to its empty state, a non-cooperative unbinding rate β is needed to prevent a singularity at the full state. For the simulations presented in the main text this addition is not necessary – any practical simulation time will never lead even near such a full state, if not started in its vicinity. Measured fluctuations in simulations cannot sample this tail of the distribution, as we will see in the ensuing results: distributions of synaptic size decay fast enough in their variable Θ. However, the stationary distributions in Eq. (S6) effectively includes an infinite time limit and thus requires at least an infinitesimally small β. This can also be observed by inspecting the definition of the unbinding rate, $k_{\text{off}} = \lambda_{\text{off}}(1-\Theta)+\beta = -\lambda_{\text{off}}\Theta + (\lambda_{\text{off}}+\beta)$, where in the latter expression the difference for small β is negligible. We are aiming to explore the effects of cooperative rates, which need to be much larger than non-cooperative rates in order to lead to the skewed distributions we want to explain.

Stationary synaptic size distributions can be calculated for each one of the three models using (S6). We compared the solutions arising from the Fokker-Planck equation with those obtained from stochastic simulations, for several cases of the different models, as shown in Fig. SI4. Both methods show a Gaussian-like symmetric distribution for the Langmuir model and for the contact process in the super-critical regime. Both exhibit a slightly skewed distribution with mean near zero for the contact process in the sub-critical regime with weak spontaneous binding. Finally, both show also similar results for the bidirectional cooperativity model. This agreement serves as a control that validates the results of the simulations shown the main text. In particular, it indicates that the skewness obtained for the bidirectional cooperativity model stems from the model itself rather than from irrelevant factors of the simulations such as boundary effects.

To analyze the stationary distribution $\mathbb{P}_\star$ in more detail, we use the most general probabilities per unit time $k_{\text{on}} = \lambda_{\text{on}}\Theta + \alpha$, $k_{\text{off}} = \lambda_{\text{off}}(1-\Theta)+\beta$, allowing to treat all models simultaneously. The various models emerge as different limits of parameter values of this general form (compare to Eqs. (S3)). To proceed analytically, we carry out the integral in the first term of (S6). Its general structure contains three terms,

$$\int_\Theta \frac{A(\Theta)-B(\Theta)}{A(\Theta)+B(\Theta)} = C_{\text{lin}}\Theta + C_{\log}\log\big(Q(\Theta)\big) + C_{\text{at}}\,\text{arctanh}\big(K(\Theta)\big) \ , \tag{S7}$$

where $Q(\Theta)$ and $K(\Theta)$ are low-order polynomials in Θ. These polynomials are specified for the different models in Table 1. Depending if one (or more) of the four parameters (λ_{on} and λ_{off}, α, or β) are zero, some of these terms in (S7) may vanish.

These expressions allow us to identify relevant parameters combinations, which will determine the moments and shape of the steady state distributions. These are the averages and differences of the cooperative and non-cooperative binding and unbinding, respectively:

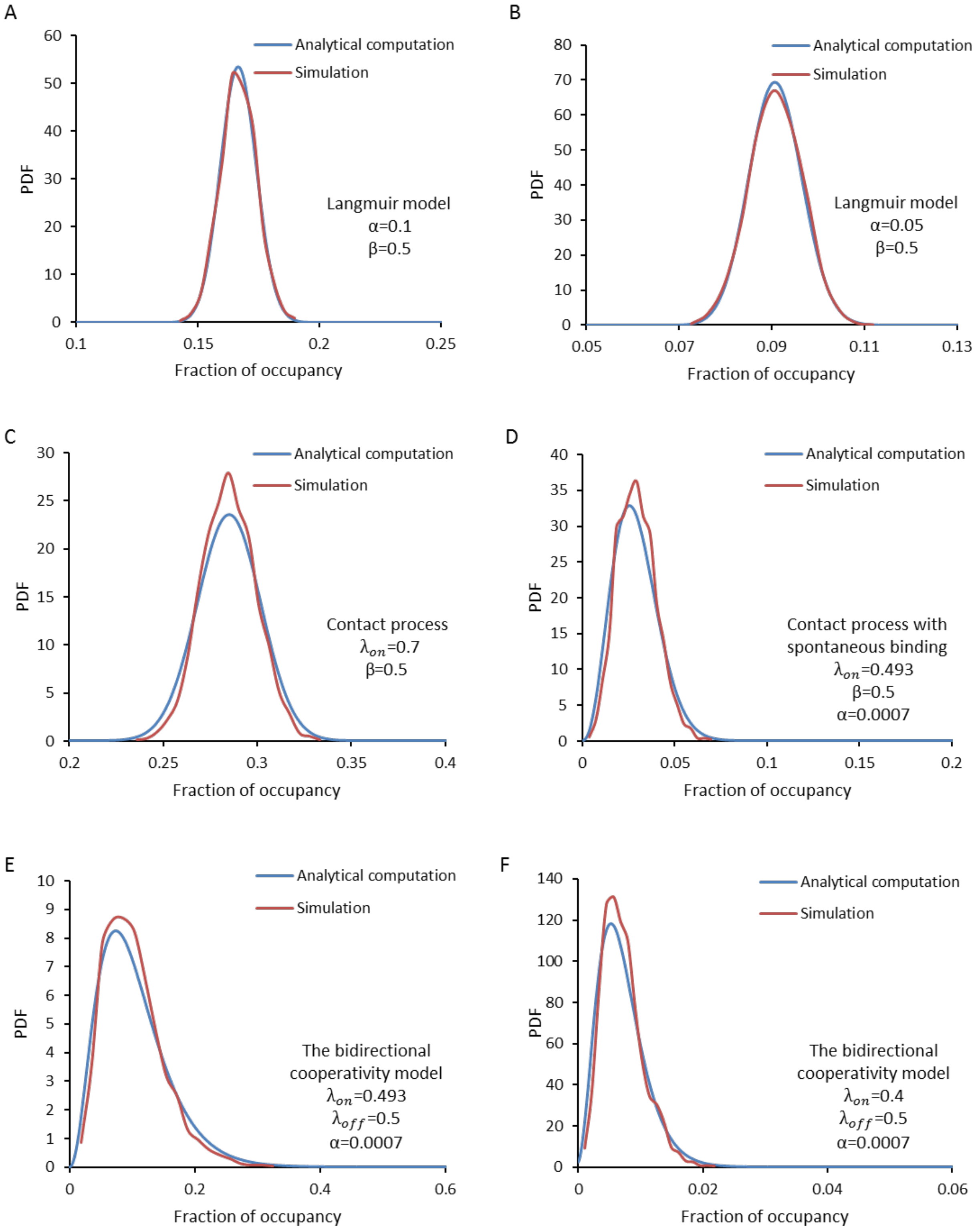

Figure SI4: **Comparison of analytic results and simulations.** Synaptic size distributions obtained from Monte Carlo simulations and master equation-based analysis of (A,B) the Langmuir model, (C,D) the contact process and (E,F) the bidirectional cooperativity model. In the three models, the analytical and the numerical distributions overlap. In the numerical simulations, the binding and unbinding rates depended on the entire occupancy of the lattice, ie. they mimick the global interaction.

λ_{on}	α	λ_{off}	β	C_{lin}	C_{log}	$Q(\Theta)$	C_{at}	$K(\Theta)$
				$\frac{\delta_1}{\mu_1}$	$\frac{\mu_1\mu_2-\delta_1\delta_2}{2\mu_1^2}$	$2\mu_1\Theta(1-\Theta)$<br>$+(\mu_2+\delta_2)(1-\Theta)$<br>$+(\mu_2-\delta_2)\Theta$	$\frac{\delta_1(\mu_1\mu_2+\delta_2^2)-\delta_2\mu_1(\mu_1+\mu_2)}{\mu_1^2\sqrt{\mu_1(\mu_1+2\mu_2)+\delta_2^2}}$	$\frac{\mu_1(2\Theta-1)+\delta_2}{\sqrt{\mu_1(\mu_1+2\mu_2)+\delta_2^2}}$
0				-1	$\frac{\mu_2-\delta_2}{2\mu_1}$		$-\frac{(\mu_1+\delta_2)(\mu_2+\delta_2)}{\mu_1\sqrt{\mu_1(\mu_1+2\mu_2)+\delta_2^2}}$	$\frac{\mu_1(2\Theta-1)+\delta_2}{\sqrt{\mu_1(\mu_1+2\mu_2)+\delta_2^2}}$
	0			$\frac{\delta_1}{\mu_1}$	$\frac{\mu_2(\mu_1+\delta_1)}{\mu_1^2}$	$2\mu_1(1-\Theta)$<br>$+(\mu_2-\delta_2)$	0	
		0		1	$\frac{\mu_2-\delta_2}{2\mu_1}$		$\frac{(\mu_1-\delta_2)(\mu_2-\delta_2)}{\mu_1\sqrt{\mu_1(\mu_1+2\mu_2)+\delta_2^2}}$	
			0	$\frac{\delta_1}{\mu_1}$	$\frac{\mu_2(\mu_1+\delta_1)}{\mu_1^2}$	$2\mu_1\Theta+2\mu_2$	0	
	0		0	$\frac{\delta_1}{\mu_1}$	0		0	
	0	0		1	$\frac{2\mu_2}{\mu_1}$	$2\mu_1(1-\Theta)+2\mu_2$	0	
0			0	-1	$-\frac{2\mu_1}{\mu_2}$	$2\mu_1\Theta+2\mu_2$	0	
0		0		$\frac{\mu_2}{\delta_2}$	$\frac{\mu_2^2-\delta_2^2}{\delta_2^2}$		0	

Table 1: **Coefficients and polynomials for the solution of the integral in (S7).** Only parameters with a values of zero are indicated in the first four columns, without an entry these parameters are assumed to be larger than zero.

$$\mu_1 = (\lambda_{\text{on}}+\lambda_{\text{off}})/2\,, \qquad \mu_2 = (\alpha+\beta)/2\,,$$
$$\delta_1 = (\lambda_{\text{on}}-\lambda_{\text{off}})/2\,, \qquad \delta_2 = (\alpha-\beta)/2\,.$$

These are the combinations which appear in the analytic solutions of the steady-state distribution. Table 1 below lists the solutions to the integral (S7) for all possible combinations of neglecting zero, one, or two of the original parameters, already using this transformation. These combinations are therefore natural candidates to be used to characterize the different behaviors in parameter space.

2.3 Analysis of the bidirectional cooperativity model using the Fokker-Planck solution

Moments in parameter space. Using the closed-form solution formulated in Eqs. (S6), (S7), and using the specific form for the bidirectional cooperativity model, one may scan the space of parameters efficiently and compute the moments of the steady-state distribution. Fig. SI5 shows the variance and skewness of the steady-state solution in a two-dimensional projection in parameter space. We choose two dimensionless parameter combinations based on the general solutions in table 1 and on the results of stochastic simulations presented in the main text.

One direction for the projection (vertical axis in the figures) is $\mu_2/\mu_1 = (\alpha+\beta)/(\lambda_{\text{on}}+\lambda_{\text{off}})$. This parameter combination characterizes the general strength of non-cooperative binding relative to cooperative binding. This parameters affects the skewness significantly; a decrease in skewness is seen as a function of this parameter (going up in the vertical direction for all values on the horizontal axis). This is in agreement with our general conclusion on the importance of cooperative binding in generating skewed distributions; given the reasonable assumption that both cooperative and some degree of non-cooperative binding and unbinding exist, this highlights the necessity of dominance of the first over the second for skewed distributions to appear.

The second direction is $-\delta_1/\mu_1 = (\lambda_{\text{off}}-\lambda_{\text{on}})/(\lambda_{\text{off}}+\lambda_{\text{on}})$; this dimensionless parameter represents the difference between cooperative binding parameters, normalized by the total strength of cooperative binding. In agreement with the more restricted parameter range spanned by the stochastic simulations, the calculation reveals that the difference between the cooperative parameters affects the skewness slightly and only very close to the phase transition a sharp decrease in skewness is observed (Fig. SI5B).

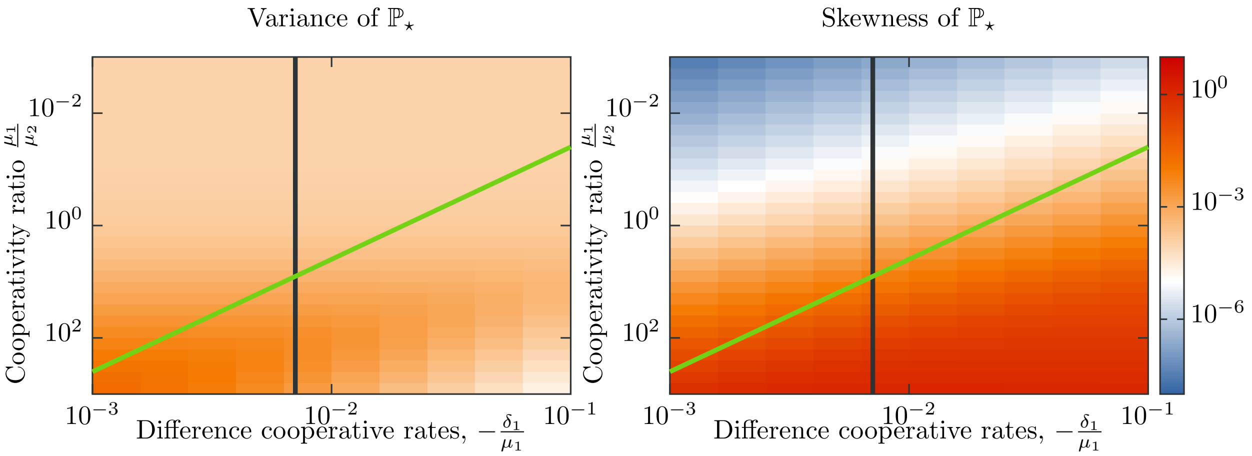

Figure SI5: **Variance and skewness of the stationary distribution in the bidirectional global cooperativity model.** Parameters are transformed such that cooperative rates (λ_{on} and λ_{off}) and non-cooperative rates (α and β) are expressed by their means and differences, see definitions in Eqs. (2.2). (A) For a small cooperativity ratio μ_1/μ_2 (non-cooperative rates are much larger than cooperative rates), we find an almost constant variance of the stationary synaptic size distribution, and its shape is close to Gaussian. This fact can also be seen in the very small values of skewness shown in (B). If cooperative rates are large, the actual value depends non-monotonically on various parameter combinations. In general, skewed distributions are obtained when cooperative are larger than non-cooperative rates (see panel (B)). Both panels use $\delta_2 = 0$, such that both non-cooperative rates are assumed identical, $\alpha = \beta$. Relaxing this assumption, we find more complicated (non-monotonic) dependence for the exact value for the moments, although the general trend is similar to the shown behavior. The green line indicates equality in the condition given by Eq. (S20), skewed distributions are found below this line. The black line shows the parameter scan presented in Fig. 7 of the main text – deviations from the roughly Gaussian distribution are clearly visible below the crossing of these two colored lines.

Analytic condition for skewed distributions. For the full set of parameters $(\lambda_{\rm on}, \lambda_{\rm off}, \alpha, \beta > 0)$, the polynomial $Q(\Theta)$ in (S7) is given by $Q = A + B$. Thus, we can write the stationary probability distribution of occupied sites as product of three terms,

$$\mathbb{P}_\star\big[\Theta\big] = cE(\Theta)BS(\Theta)AT(\Theta) \; , \tag{S8}$$

which arise from the three terms in the integral computed above, (S7), while c is the normalization constant ($c = \exp(c')$ from Eq. (S6) above). Here, the expression for $BS(\Theta)$ also contains the logarithmic term in Eq. (S6). Explicitly, we have

$$\begin{aligned} E(\Theta) &= \exp\big(2MC_{\rm lin}\Theta\big) \; , && \text{(S9a)} \\ BS(\Theta) &= \big(A(\Theta) + B(\Theta)\big)^{2MC_{\log}-1} \; , && \text{(S9b)} \\ AT(\Theta) &= \left(\frac{1+K(\Theta)}{1-K(\Theta)}\right)^{MC_{\rm at}} \; . && \text{(S9c)} \end{aligned}$$

In the following, we analyze the shape and characteristic length scales for each of those terms. It will be seen that $BS(\Theta)$ (”Bell-Shaped”) is a symmetric Gaussian-like curve, whereas $E(\Theta)$ is exponential-like and $AT(\Theta)$ a skewed function (originating from the arctanh expression). Therefore, within the product of symmetric and skewed function, relative length-scales are important, and in particular the shape of the product will be dominated by the factor with the shortest length-scale. In this context, we define length scales as a change in Θ, which also changes the value of these functions significantly. This allows to check if any of these terms is dominant and determines the shape of the stationary distribution $\mathbb{P}_\star[\Theta]$ on its own. Each term is treated separately in a paragraph below.

First, we inspect the central term $BS(\Theta)$ in more detail. We find that $A + B$ is a parabola curving downward with a single maximum. Moreover, for any realistic parameter combination, we find $A + B > 0$ for all $\Theta \in \big[0; 1\big]$, such that $(A + B)$ can be “normalized” and seen as a distribution itself. Including the exponent $2MC_{\log} - 1$, we find $BS(\Theta)$ is symmetric with respect to its mode, and looks in general bell-shaped. The mode $\Theta_{\rm md}$ of this bell-shaped curve $(A + B)^{2MC_{\log}-1}$ is given by

$$\Theta_{\rm md} = \frac{1}{2} - \frac{\delta_2}{2\mu_1} \; . \tag{S10}$$

Its value is important for estimating scales of the other terms in the full distribution $\mathbb{P}_\star[\Theta]$: we are interested in how the slopes of this bell-shape are changed near its peak.

Knowing the general shape of $BS(\Theta)$, we estimate its length scale by using the variance of the peak,

$$L_{\rm BS} \approx \sqrt{{\rm Var}\left[(A + B)^m\right]} \; , \tag{S11}$$

where $m = 2MC_{\log} - 1$. For $m < 0$, the bell-shaped parabola flips to an U-shaped curve, shifting almost all mass of $(A + B)^m$ to the boundaries. In this case, the “variance” is constant and close to 1 as the U-shape spans the entire interval – we find that (S11) is independent of m. More relevant is the case $m > 0$: Using m as an auxiliary variable, find that ${\rm Var}\big[(A + B)^m\big] \approx {\rm Var}\big[A + B\big]/m$ holds to a very good extent (up to a numerical constant of ≈ 0.8). For large system sizes M (above a certain critical size $M_{\rm crit}$) we know that m and M are linearly dependent (see Fig. SI6) and thus ${\rm Var}\big[(A + B)^m\big] \approx {\rm Var}\big[A + B\big]/\big(M/M_{\rm crit}\big)$. In particular, at $m = 1$ the scaling between the variances is the trivial identity. Thus, we can set $1 = m$ using $m = (2M_{\rm crit}C_{\log} - 1)$, from which we find $M_{\rm crit} = C_{\log}^{-1}$. The length scale $L_{\rm BS}$ of the bell-shaped part $BS(\Theta)$ is hence

$$L_{\rm BS} \approx \sqrt{\frac{{\rm Var}\big[A + B\big]}{C_{\log}M}} \; . \tag{S12}$$

In addition, we can carry out the integrals $\int d\Theta \; \Theta^k(A + B)$ with $k = 0, 1, 2$ to obtain the variance of the “distribution” $(A + B)$ at $m = 1$, leading to

$${\rm Var}\big[A + B\big] = \frac{1}{20}\frac{(\mu_1 + 3\mu_2)(\mu_1 + 5\mu_2) - 5\delta_2^2}{(\mu_1 + 3\mu_2)^2} \; .$$

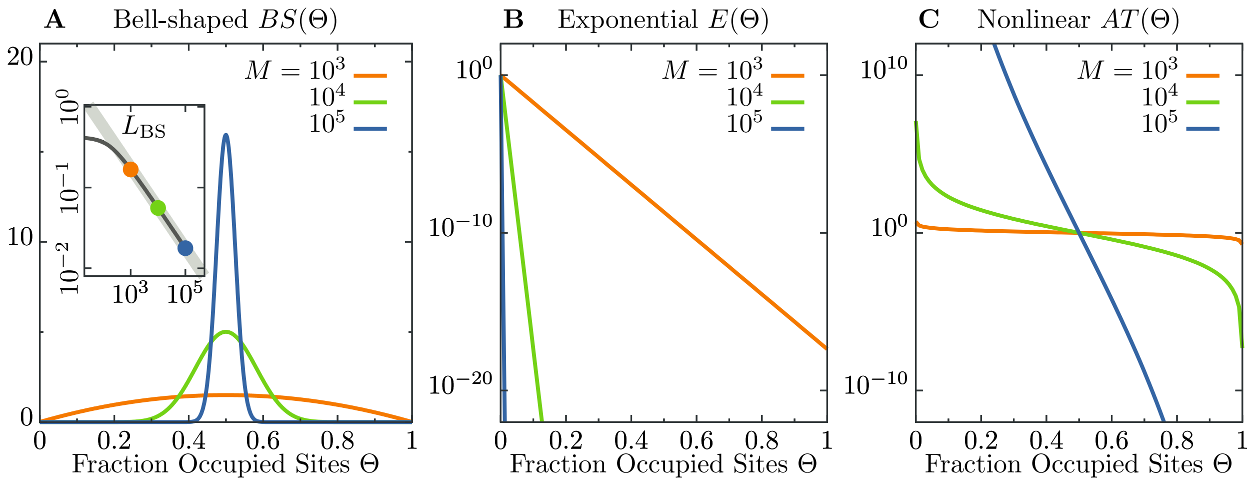

Figure SI6: **Shapes of the different terms in the stationary distribution depending on system size.** For illustration, we chose $\delta_2 = 0$, which sets the mode of $BS(\Theta)$ at $\Theta_{md} = 1/2$, although results also hold for general values. (A) The bell-shaped term $BS(\Theta)$ is very close to Gaussian, its width scales with system size $M^{-1/2}$ (light gray line in inset). Colored dots in inset indicate values of L_{BS} for shown shapes of $BS(\Theta)$, dark gray line indicates L_{BS} for intermediate values of M. (B) Exponential term $E(\Theta)$. (C) The nonlinear term $AT(\Theta)$ generated by exponentiating the arctanh-expression in Eq. (S7) is very close to exponential at the mode of the bell-shaped term, but significantly deviates at the boundaries.

Combining these expressions, we find that the length scale (ie. width) of the symmetric, bell-shaped part $BS(\Theta)$ is given by

$$L_{BS} \approx \frac{1}{\sqrt{10M}} \sqrt{\frac{\mu_1^2\big((\mu_1 + 3\mu_2)(\mu_1 + 5\mu_2) - 5\delta_2^2\big)}{\big(\mu_1\mu_2 - \delta_1\delta_2\big)\big(\mu_1 + 3\mu_2\big)^2}}\ . \tag{S13}$$

Next we check the second term in the product $E(\Theta)$, given by a simple exponential, cf. (S9a). Thus, the length scale L_E can be directly read from the expression itself, which is of the form $E(\Theta) \sim \exp\big(\Theta/L_E\big)$. Consequently, we arrive at

$$L_E = 1/\big(2MC_{lin}\big)\ ,$$

or, using the explicit expression for C_{lin} in Tbl. 1,

$$L_E = \frac{1}{2M}\frac{\mu_1}{\delta_1}\ . \tag{S14}$$

For the last term in the product, $AT(\Theta)$, numerical evaluation shows that it can change its value quite significantly over the range of Θ. However, it is approximately exponential for values of Θ close to Θ_{md} given by Eq. (S10) (see Fig. SI6C). Thus, we generalize the length scale definition from the previous paragraph on $E(\Theta)$ by setting

$$L_{AT} \equiv \Big(\partial_\Theta \log AT(\Theta)\big|_{\Theta_{md}}\Big)^{-1}\ , \tag{S15}$$

which again assumes that in the vicinity of Θ_{md} we have $AT(\Theta) \sim \exp\big(\Theta/L_{AT}\big)$. Notice that this expression generates exactly the length scale L_E in (S14) when applied to $E(\Theta)$ in (S9a).

After inserting the expression for $AT(\Theta)$ from (S9c) we obtain

$$L_{AT} = \frac{1}{4MC_{at}}\frac{1 - K(\Theta)^2}{K'(\Theta)}\bigg|_{\Theta_{md}}\ .$$

Referencing the results listed in Tbl. 1 and inserting the mode $\Theta_{md} = \frac{1}{2} - \frac{\delta_2}{2\mu_1}$, cf. (S10), we get

$$L_{AT} = \frac{1}{8M}\frac{2\mu_1\delta_2^2 + 2\mu_1^3 + \mu_1^2\mu_2}{\delta_1\delta_2^2 + \delta_1\mu_1\mu_2 - \delta_2\mu_1^2 - \delta_2\mu_1\mu_2}\ . \tag{S16}$$

Summarizing the length-scale analysis, we found that $BS(\Theta)$ is a symmetric, bell-shaped curve centered around Θ_{md}. Moreover, both $E(\Theta)$ and $AT(\Theta)$ introduce an exponential skew to the stationary distribution of synaptic sizes. Thus, if the latter terms dominate, we will find highly skewed distributions, while as long as $BS(\Theta)$ dominates, we see an almost Gaussian distribution of synaptic sizes.

The dominant term in the stationary solution corresponds to the smallest length scale. However, in order to properly compare symmetric and skewed parts of the distribution, we have to combine the two terms $E(\Theta)$ and $AT(\Theta)$. To this end, note that applying the length scale definition of (S15) to the product $E(\Theta)AT(\Theta)$ leads to the relation $L_{\text{E,AT}}^{-1} = L_{\text{E}}^{-1} + L_{\text{AT}}^{-1}$. Evaluating this relation yields

$$L_{\text{E,AT}}^{-1} = 2M \frac{\delta_1 \left(2\mu_1^2 + 5\mu_1\mu_2\right) + \delta_2 \left(5\delta_1\delta_2 - 4\mu_1^2 - 4\mu_1\mu_2\right)}{\mu_1 \left(\mu_1^2 + \mu_1\mu_2 + \delta_2^2\right)} . \tag{S17}$$

Now we can compare L_{BS} and $L_{\text{E,AT}}$. The smaller of these scales will dominate the stationary solution $\mathbb{P}_\star[\Theta]$. If L_{BS} is smaller we have a roughly Gaussian shape. Contrary, if $L_{\text{E,AT}}$ is smaller, we find $\mathbb{P}_\star[\Theta]$ to be a very fast decaying distribution, with a large skew. The direct comparison of the full expressions is rather cumbersome to analyze,

$$\begin{aligned} L_{\text{E,AT}} \quad &\ll \quad L_{\text{BS}} \qquad \Leftrightarrow \\ \frac{1}{2M} \frac{\mu_1 \left(\mu_1^2 + \mu_1\mu_2 + \delta_2^2\right)}{\delta_1 \left(2\mu_1^2 + 5\mu_1\mu_2\right) + \delta_2 \left(5\delta_1\delta_2 - 4\mu_1^2 - 4\mu_1\mu_2\right)} \quad &\ll \quad \frac{1}{4\sqrt{M}} \sqrt{\frac{\mu_1^2 \left((\mu_1 + 3\mu_2)(\mu_1 + 5\mu_2) - 5\delta_2^2\right)}{\left(\mu_1\mu_2 - \delta_1\delta_2\right)\left(\mu_1 + 3\mu_2\right)^2}} \end{aligned} \tag{S18}$$

In order to gain more insight, we chose a particularly simple limit of (S18). We set $\delta_2 = 0$, such that the two non-cooperative rates α and β are equal (or at least *very* similar and their distance is small). The two length scales become (ignoring $\mathcal{O}(1)$ numerical constants):

$$L_{\text{BS}} \sim \frac{1}{\sqrt{M}} \sqrt{\frac{\mu_1}{\mu_2}} , \tag{S19a}$$

$$L_{\text{E,AT}} \sim \frac{1}{M} \left| \frac{\mu_1}{\delta_1} \right| . \tag{S19b}$$

A few conclusions can be drawn by comparing (S19). When the system is large, $M \gg 1$, we will usually arrive in a regime where the distribution of synaptic sizes is skewed. If the cooperative rates, λ_{on} and λ_{off}, are a lot larger than the non-cooperative rates, α and β, we also expect rather skewed distributions. Conversely, very similar cooperative rate constants, λ_{on} and λ_{off}, favor Gaussian shaped distribution.

With the scalings in Eq. (S19) at hand, we can also reevaluate the condition for skewed distributions, Eq. (S18). In particular, the reduced inequality becomes

$$\frac{|\lambda_{\text{on}} - \lambda_{\text{off}}|}{\lambda_{\text{on}} + \lambda_{\text{off}}} \gg \frac{1}{\sqrt{M}} \sqrt{\frac{\alpha + \beta}{\lambda_{\text{on}} + \lambda_{\text{off}}}} . \tag{S20}$$

When this inequality does not hold, we expect Gaussian distribution of synaptic sizes.

With this condition, Eq. (S20), at hand we can revisit results derived earlier, in particular compare it to the condition obtained from the mean field analysis: There, we derived $\alpha \leq \lambda_{\text{off}} - \lambda_{\text{off}}$ (without including β yet) as indicating parameter values, where the dynamics will does not run into its absorbing state of a full scaffold. Using the default value $M \sim \mathcal{O}(10^3)$ for the system size, which is roughly equal to $\mu_2^{-1} = 1/(\alpha + \beta)$, both of these conditions actually restrict parameters for stable and skewed distributions to similar ranges of parameters space: Adding large enough cooperative rates is already sufficient to explain observed synaptic size dynamics.
